# Practice-Level Variation in Molecular Testing and Use of Targeted Therapy for Patients With Non–Small Cell Lung Cancer and Colorectal Cancer

**DOI:** 10.1001/jamanetworkopen.2023.10809

**Published:** 2023-04-28

**Authors:** Thomas J. Roberts, Kenneth L. Kehl, Gabriel A. Brooks, Lynette Sholl, Alexi A. Wright, Mary Beth Landrum, Nancy L. Keating

**Affiliations:** 1 Division of Population Sciences, Dana-Farber Cancer Institute, Boston, Massachusetts; 2 Department of Medicine, Massachusetts General Hospital, Boston; 3 Section of Medical Oncology, Dartmouth Hitchcock Medical Center, Lebanon, New Hampshire; 4 Department of Pathology, Brigham and Women’s Hospital, Boston, Massachusetts; 5 Department of Health Care Policy, Harvard Medical School, Boston, Massachusetts; 6 Division of General Internal Medicine, Brigham and Women’s Hospital, Boston, Massachusetts

## Abstract

**Importance:**

All patients with newly diagnosed non–small cell lung cancer (NSCLC) and colorectal cancer (CRC) should receive molecular testing to identify those who can benefit from targeted therapies. However, many patients do not receive recommended testing and targeted therapies.

**Objective:**

To compare rates of molecular testing and targeted therapy use by practice type and across practices.

**Design, Setting, and Participants:**

This cross-sectional study used 100% Medicare fee-for-service data from 2015 through 2019 to identify beneficiaries with new metastatic NSCLC or CRC diagnoses receiving systemic therapy and to assign patients to oncology practices. Hierarchical linear models were used to characterize variation by practice type and across practices. Data analysis was conducted from June 2019 to October 2022.

**Exposures:**

Oncology practice providing care.

**Outcomes:**

Primary outcomes were rates of molecular testing and targeted therapy use for patients with NSCLC and CRC. Secondary outcomes were rates of multigene testing for NSCLC and CRC.

**Results:**

There were 106 228 Medicare beneficiaries with incident NSCLC (31 521 [29.7%] aged 65-69 years; 50 348 [47.4%] female patients; 2269 [2.1%] Asian, 8282 [7.8%] Black, and 91 215 [85.9%] White patients) and 39 512 beneficiaries with incident CRC (14 045 [35.5%] aged 65-69 years; 17 518 [44.3%] female patients; 896 [2.3%] Asian, 3521 [8.9%] Black, and 32 753 [82.9%] White patients) between 2015 and 2019. Among these beneficiaries, 18 435 (12.9%) were treated at National Cancer Institute (NCI)–designated centers, 8187 (5.6%) were treated at other academic centers, and 94 329 (64.7%) were treated at independent oncology practices. Molecular testing rates increased from 74% to 85% for NSCLC and 45% to 65% for CRC. First-line targeted therapy use decreased from 12% to 8% among patients with NSCLC and was constant at 5% for patients with CRC. For NSCLC, molecular testing rates were similar across practice types while rates of multigene panel use (13.2%) and targeted therapy use (16.6%) were highest at NCI-designated cancer centers. For CRC, molecular testing rates were 3.8 (95% CI: 1.2-6.5), 3.3 (95% CI, 0.4-6.1), and 12.2 (95% CI, 9.1-15.3) percentage points lower at hospital-owned practices, large independent practices, and small independent practices, respectively. Rates of targeted therapy use for CRC were similar across practice types. After adjusting for patient characteristics, there was moderate variation in molecular testing and targeted therapy use across oncology practices.

**Conclusions and Relevance:**

In this cross-sectional study of Medicare beneficiaries, molecular testing rates for NSCLC and CRC increased in recent years but remained lower than recommended levels. Rates of targeted therapy use decreased for NSCLC and remained stable for CRC. Variation across practices suggests that where a patient was treated may have affected access to recommended testing and efficacious treatments.

## Key Points

**Question:**

How do molecular testing and targeted therapy use for patients with colorectal cancer (CRC) and non–small cell lung cancer (NSCLC) vary across oncology practices?

**Findings:**

In this cross-sectional study of 145 740 Medicare beneficiaries, rates of molecular testing for NSCLC were similar across practice types, but multigene panel and targeted therapy use were highest at National Cancer Institute (NCI)–designated cancer centers. Among patients with CRC, molecular testing was highest at NCI-designated cancer centers and academic centers, and targeted therapy use was similar across practice types.

**Meaning:**

In this study, use of recommended molecular testing and targeted therapies varied by practice type among patients with NSCLC and CRC.

## Teaser

This cross-sectional study compares rates of molecular testing and targeted therapy among patients with non–small cell lung cancer (NSCLC) and colorectal cancer (CRC) use by practice type and across practices.

## Introduction

Targeted therapies have substantially improved outcomes for some patients with non–small cell lung cancer (NSCLC) and colorectal cancer (CRC).^1,2,3,4^ Approximately 20% of patients with metastatic NSCLC have somatic variants for which there are efficacious targeted therapies.^5^ For metastatic CRC, more than 50% of patients have somatic variant that could inform the choice of first-line therapy.^6,7^

Since 2011, the standard of care for metastatic nonsquamous NSCLC included somatic molecular testing to identify biomarkers to guide treatment selection. Since 2016, all patients with metastatic NSCLC, regardless of histology, were recommended to receive programmed cell death ligand 1 (PD-L1) testing, and by 2019, recommendations included testing for *EGFR*, *ALK*, *ROS1*, *BRAF*, and *NTRK* alterations.^8^ Similarly, molecular testing has been recommended for patients with newly diagnosed metastatic CRC since 2009. By 2019, recommendations for CRC included testing for *KRAS*, *NRAS*, and *BRAF* alterations and for microsatellite instability (MSI).^9,10^

There are multiple methods to test for these alterations, and the most common method varies by biomarker. For example, *EGFR* variants can be identified by polymerase chain reaction (PCR), next-generation sequencing (NGS)–based multigene panels, and less commonly immunohistochemistry (IHC). *ALK* rearrangements are usually identified by florescent in-situ hybridization (FISH), NGS, or IHC. Molecular testing often involves multiple assays, although multigene panels can identify most commonly encountered alterations in a single assay.^11^ PD-L1 expression can only be done by IHC and is therefore not included in multigene panels.

Low rates of molecular testing have been associated with lower use of targeted therapies among patients with NSCLC, inappropriate use of targeted therapies among patients with CRC, and worse overall survival.^12,13,14^ Despite recommendations of universal testing, rates of *EGFR* and *ALK* testing were 48% to 68% among patients with metastatic NSCLC,^15,16,17^ and rates of testing for *RAS*, *BRAF*, and MSI were 40% to 60% in patients with metastatic CRC.^12,18,19^ Similarly, only 9.8% of patients at community oncology practices received *EGFR/ALK* targeted therapy.^20^ Rates of molecular testing and targeted therapy use vary by race, insurance type, and geography.^15,21^ It is unclear to what extent molecular testing and targeted therapy use vary across oncology practices. In this study, we aimed to describe trends in molecular testing and targeted therapy use among Medicare beneficiaries over time, to characterize how rates vary by practice type and patient characteristics, and to assess variation in molecular testing and targeted therapy use across oncology practices.

## Methods

### Data and Study Population

We used Medicare inpatient, outpatient, supplier, and durable medical equipment claims from 2015 through 2019 for 100% of Medicare fee-for-service beneficiaries aged 65 years and older. The Harvard Medical School institutional review board approved this study and the need for informed consent because the secondary analysis involved no more than minimal risk to the individuals in the data set. This study followed the Strengthening the Reporting of Observational Studies in Epidemiology (STROBE) reporting guidelines.

Among patients with at least 2 outpatient office visits or 1 inpatient admission for lung, colon, or rectal cancer, defined based on *International Classification of Diseases, Ninth Revision* (*ICD-9*) or *International Statistical Classification of Diseases and Related Health Problems, Tenth Revision* (*ICD-10*) diagnosis codes, we identified patients with at least 1 claim for systemic therapy for these cancers (eTables 1 and 2 in Supplement 1). We included patients continuously enrolled for 180 days before and after the first systemic therapy claim. When assessing targeted therapy use, we restricted the analyses to patients with Medicare Part D coverage for 180 days after treatment initiation.^22^

To identify patients with metastatic NSCLC, we excluded patients with claims for irinotecan, topotecan, or carboplatin/cisplatin plus etoposide within 5 days of the first treatment, as these patients likely had small cell lung cancer. We used a clinical algorithm to select patients with metastatic NSCLC by excluding patients with claims for lung cancer resections (eTable 3 in Supplement 1) in the 180 days after treatment initiation or radiation therapy (eTable 4 in Supplement 1) in the 30 days after treatment initiation.^23^ For CRC, we excluded patients with claims for cancer resection (eTable 5 in Supplement 1) in the 180 days after treatment initiation. To identify new or newly recurrent cancers, we only included patients with a period of 180 days without a claim for lung or colorectal cancer preceding treatment initiation (patients with metastatic cancer usually have encounters at more frequent intervals). Because patients do not typically start systemic therapy at the first encounter for cancer, we began the 180-day lookback period at an estimated diagnosis date, which we estimated as the date of the first encounter for lung or colorectal cancer in the 90 days before the first systemic therapy claim.

### Outcomes

The primary outcomes were the percentage of patients with NSCLC and CRC who had at least 1 claim for molecular testing between 90 days before through 60 days after treatment initiation and the percentage of patients with a claim for targeted therapy within 30 days of treatment initiation. Molecular testing claims included predictive assays that could inform targeted therapy or immunotherapy selection (eTable 6 in Supplement 1). Secondary outcomes were the percentage of patients with NSCLC and CRC with claims for multigene panels. We also assessed rates of immunotherapy use during the study period. Codes within each category are detailed in eTables 1, 2, and 6 in Supplement 1.

### Independent Variables

Patients were assigned to oncology practices based on the plurality of office visits with oncologists in the 180 days following treatment initiation.^24^ We identified practices affiliated with academic medical centers^25^ and National Cancer Institute (NCI)–designated cancer centers as of 2018.^26^ We identified other hospital-owned practices in each year if more than 90% of the claims were billed from a hospital outpatient department.^27^ The remaining practices were categorized as small (≤5 medical oncologists billing to the practice) or large (>5 medical oncologists) independent practices.

We used patient age, sex, and race and ethnicity as reported in Medicare enrollment data. Race and ethnicity were categorized as Asian, Hispanic, non-Hispanic Black, non-Hispanic White, or other (Pacific Islander, American Indian or Alaska Native, other, unknown) based on the Research Triangle Institute race variable.^28^ Dual eligibility for Medicare and Medicaid was determined based on the month of treatment initiation. We characterized comorbidity using the Klabunde modification of the Charlson Comorbidity Index based on claims during the 180 days before treatment initiation.^29^ We defined rural vs urban zip code of residence using the 2010 Rural-Urban Commuting Area codes and characterized zip code–level median household income and high school graduation rates using 2015 American Community Survey data.^30,31^ We characterized CRC tumors as left-sided, right-sided, or rectal using *ICD-10* codes (eTable 7 in Supplement 1). Variables were categorized as in Table 1.

**Table 1.** Characteristics of the Study Population

Characteristic	Patients, No. (%)	
	NSCLC	Colorectal cancer
Age, y		
65-69	31 521 (29.7)	14 045 (35.5)
70-74	29 350 (27.6)	9154 (23.2)
75-79	24 224 (22.8)	7296 (18.5)
80-84	14 118 (13.3)	5125 (13.0)
≥85	7015 (6.6)	3892 (9.9)
Sex		
Female	50 348 (47.4)	17 518 (44.3)
Male	55 880 (52.6)	21 994 (55.7)
Race and ethnicity		
Asian	2269 (2.1)	896 (2.3)
Black (non-Hispanic)	8282 (7.8)	3521 (8.9)
Hispanic	992 (0.9)	617 (1.6)
White (non-Hispanic)	91 215 (85.9)	32 753 (82.9)
Other^a^	3470 (3.3)	1725 (4.4)
Urban residence		
Urban	83 584 (78.7)	30 662 (77.6)
Large rural	11 783 (11.1)	4405 (11.1)
Small rural	6249 (5.9)	2569 (6.5)
Isolated	4612 (4.3)	1876 (4.7)
Median income^b^		
≥$80 000	16 460 (15.5)	6183 (15.6)
$60 000 to <$80 000	22 519 (21.2)	8163 (20.7)
$40 000 to <$60 000	44 851 (42.2)	16 488 (41.7)
<$40 000	20 696 (19.5)	7916 (20.0)
Unknown	1702 (1.6)	762 (1.9)
High school graduation rate^b^		
≥93%	29 461 (27.7)	10 762 (27.2)
89%-92%	25 769 (24.3)	9156 (23.2)
83%-88%	26 268 (24.7)	9671 (24.5)
0%-82%	23 295 (21.9)	9261 (23.4)
Unknown	1435 (1.4)	662 (1.7)
Dual eligible		
Yes	16 892 (15.9)	7141 (18.1)
No	89 336 (84.1)	32 371 (81.9)
Charlson score		
0	39 960 (37.6)	22 227 (56.3)
1	28 665 (27.0)	7808 (19.8)
2	16 434 (15.5)	4411 (11.2)
≥3	21 169 (19.9)	5066 (12.8)
Practice type		
NCI-designated cancer center	13 485 (12.7)	4950 (12.5)
Other academic center	6021 (5.7)	2166 (5.5)
Other hospital-owned practice	18 277 (17.2)	6512 (16.5)
Large independent practice	53 709 (50.6)	19 798 (50.1)
Small independent practice	14 736 (13.9)	6086 (15.4)
Primary tumor site		
Left sided	NA	5684 (14.4)
Right sided	NA	8377 (21.2)
Rectal	NA	18 715 (47.4)
Unknown	NA	6736 (17.0)

Abbreviations: NA, not applicable; NCI, National Cancer Institute; NSCLC, non–small cell lung cancer.

^a^ Includes Pacific Islander, American Indian or Alaska Native, other, and unknown.

^b^ Calculated at the zip code level.

### Statistical Analysis

We used hierarchical linear regression models with patient characteristics and practice type to identify characteristics associated with molecular testing and targeted therapy use. Models were run separately for each cancer type and included all independent variables. We included practice-level random effects and estimated rates of molecular testing and targeted therapy use for each practice after adjusting for patient characteristics. For practice-level estimates, we did not include the practice type in the models because this variable may contribute to observed variation. Statistical tests were 2-sided. *P* < .05 was considered statistically significant. We did not adjust for multiplicity in these exploratory analyses but included 95% CIs for all comparisons to indicate precision of our estimates. Analyses were performed using SAS version 9.4 (SAS Institute).

For NSCLC, we performed sensitivity analyses assessing molecular testing excluding claims for IHC (IHC claims do not distinguish between predictive and diagnostic testing) and among patients who received pemetrexed or bevacizumab (likely to have nonsquamous cancers). We also assessed targeted therapy use after October 2016, when the US Food and Drug Administration (FDA) narrowed the approved indication for erlotinib maintenance to patients with *EGFR*-variant NSCLC.^8,32^

Missing data were infrequent. For patients with missing zip codes (<2%), area-level income and education were coded as unknown. Patients with missing race and ethnicity (N = 2403) were grouped with other.

## Results

### Cohort Selection

**Figure 1.**
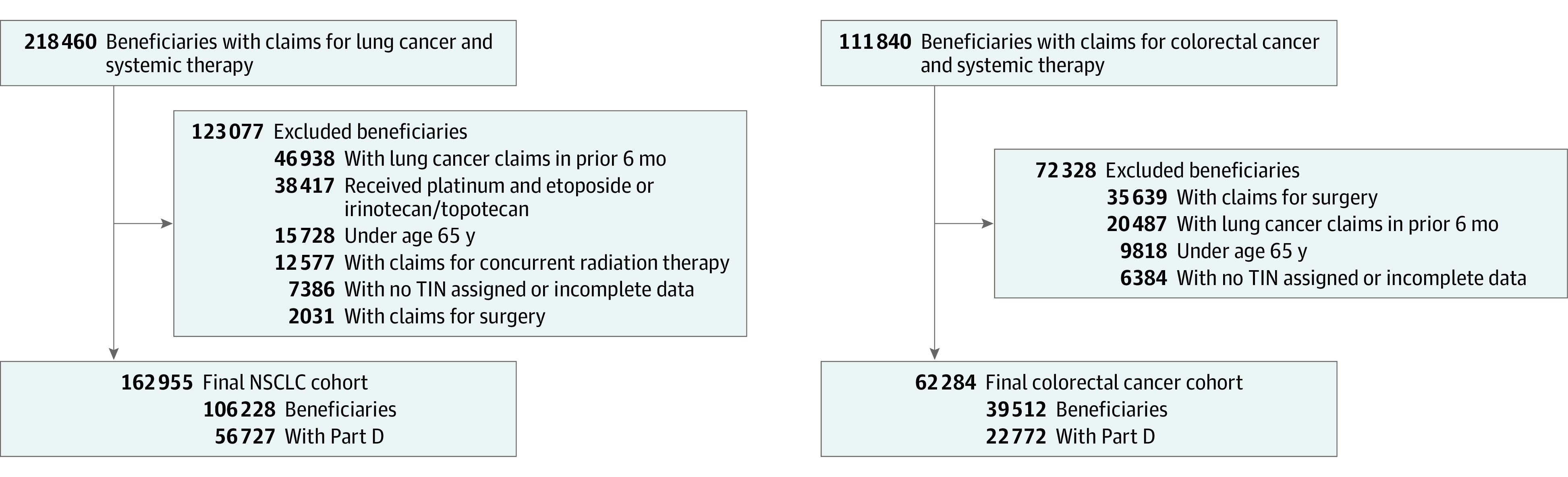
Study Flow Diagram Showing Selection of Cohorts of Patients With Newly Diagnosed Metastatic Non–Small Cell Lung Cancer (NSCLC) and Colorectal Cancer TIN indicates tax identification number.

Our final study populations included 106 228 patients with metastatic NSCLC (31 521 [29.7%] aged 65-69 years; 50 348 [47.4%] female patients; 2269 [2.1%] Asian, 8282 [7.8%] Black, and 91 215 [85.9%] White patients) and 39 512 patients with metastatic CRC (14 045 [35.5%] aged 65-69 years; 17 518 [44.3%] female patients; 896 [2.3%] Asian, 3521 [8.9%] Black, and 32 753 [82.9%] White patients) who initiated systemic therapy between July 2015 and December 2019 (Table 1). For the analysis of targeted therapy use, 56 727 patients with NSCLC and 22 772 patients with CRC with continuous Medicare Part D coverage were included (Figure 1). Approximately 13% of patients received care at NCI-designated cancer centers (18 435 [12.9%]), 6% at other academic medical centers (8187 [5.9%]), 17% at other hospital-based practices (18 277 [17.2%]), and 65% at independent oncology practices (94 329 [64.7%]). The number of patients with Part D coverage receiving systemic treatment was stable over time: approximately 3500 patients with NSCLC and 1400 patients with CRC per quarter.

### Molecular Testing

**Figure 2.**
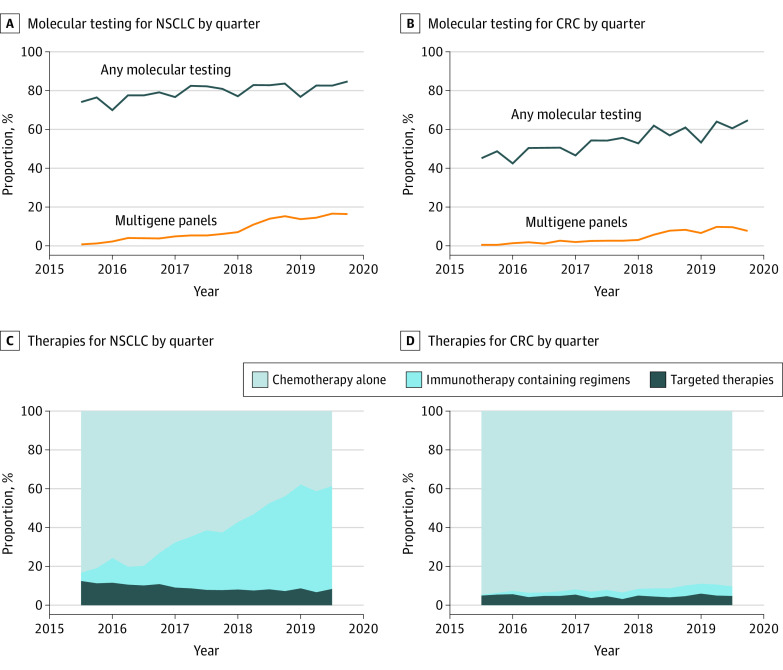
Proportion of Medicare Fee-for-Service Beneficiaries With Newly Diagnosed Metastatic Non–Small Cell Lung Cancer (NSCLC) and Colorectal Cancer (CRC) Who Received Molecular Testing and Types of Therapy Used for First-line Treatment by Quarter, 2015 to 2019 C and D, Includes only patients with Part D coverage.

Molecular testing increased over time for patients with NSCLC and CRC (Figure 2A and B). By the end of 2019, 4342 of 5129 patients with NSCLC (84.7%) had claims for at least 1 molecular test, up from 4339 of 5852 (74.1%) in 2015 (Figure 2A). Use of multigene panels increased from 42 (0.7%) in 2015 to 839 (16.4%) in 2019. Among patients with CRC (Figure 2B), molecular testing rates increased from 1038 of 2297 (45.2%) in 2015 to 1270 of 1962 (64.7%) in 2019, and use of multigene panels increased from 10 (0.4%) in 2015 to 149 (7.6%) in 2019. Excluding claims for IHC, molecular testing rates increased from 2262 of 5852 (38.7%) in 2015 to 2588 of 5129 (50.5%) in 2019 for patients with NSCLC and from 438 of 2297 (19.1%) to 560 of 1962 (28.5%) in 2019 for patients with CRC.

Table 2 shows unadjusted rates and adjusted percentage point differences for molecular testing by practice type, race and ethnicity, and dual eligibility (eTable 8 in Supplement 1). Overall, 78.9% of patients with NSCLC had a claim for molecular testing. In adjusted analyses, molecular testing rates among patients with NSCLC did not vary by practice type. Rates of multigene panel use were approximately 5 percentage points lower at all practice types compared with NCI-designated cancer centers. Compared with non-Hispanic White patients, molecular testing rates were similar among Hispanic patients and 2.7 (95% CI, 1.8-3.7) percentage points lower among Black patients. Molecular testing use was lower among dually eligible patients.

**Table 2.** Unadjusted Rates and Adjusted Percentage Point Differences of Molecular Testing and Targeted Therapy Use in Patients with Metastatic NSCLC and Colorectal Cancer^a^

Characteristic	NSCLC						Colorectal cancer					
	Any molecular testing		Multigene panels		Targeted therapy use		Any molecular testing		Multigene panels		Targeted therapy use	
	Unadjusted rate, %	Adjusted difference (95% CI), percentage point	Unadjusted rate, %	Adjusted difference (95% CI), percentage point	Unadjusted rate, %	Adjusted difference (95% CI), percentage point	Unadjusted rate, %	Adjusted difference (95% CI), percentage point	Unadjusted rate, %	Adjusted difference (95% CI), percentage point	Unadjusted rate, %	Adjusted difference (95% CI), percentage point
Practice type												
NCI-designated center	78.9	[Reference]	13.2	[Reference]	16.6	[Reference]	56.9	[Reference]	5.5	[Reference]	5.0	[Reference]
Other academic center	78.3	−1.3 (−3.7 to 1.1)	7.2	−4.7 (−6.8 to −2.6)	9.1	−4.8 (−6.4 to −3.2)	57.2	0.8 (−3.2 to 4.9)	4.8	−0.9 (−2.4 to 0.6)	6.1	1.2 (−0.4 to 2.7)
Other hospital-owned	79.3	−0.4 (−2.0 to 1.2)	8.3	−4.7 (−6.2 to −3.2)	8.0	−5.4 (−6.4 to −4.3)	54.4	−3.8 (−6.4 to −1.2)	4.4	−1.3 (−2.4 to −0.3)	5.0	−0.05 (−1.0 to 0.9)
Large independent	80.0	−0.01 (−1.7 to 1.7)	7.5	−5.0 (−6.5 to −3.6)	7.4	−6.4 (−7.5 to −5.2)	54.9	−3.3 (−6.1 to −0.4)	4.2	−1.8 (−2.7 to −0.8)	4.7	−0.3 (−1.4 to 0.9)
Small independent	78.0	−1.4 (−3.3 to 0.5)	6.0	−5.2 (−6.7 to −3.8)	9.5	−6.2 (−7.6 to −4.8)	46.2	−12.2 (−15.3 to −9.1)	2.5	−2.7 (−3.7 to −1.6)	3.8	−1.2 (−2.6 to 0.1)
Race and ethnicity												
White, non-Hispanic	80.2	[Reference]	8.3	[Reference]	7.4	[Reference]	54.7	[Reference]	4.2	[Reference]	4.6	[Reference]
Asian	75.5	−1.2 (−3.0 to 0.5)	8.7	−0.3 (−1.5 to 0.9)	46.6	34.7 (33.2 to 36.1)	45.3	−5.6 (−8.9 to −2.2)	3.1	−0.6 (−2.0 to 0.7)	4.5	−0.4 (−2.2 to 1.3)
Black	74.6	−2.7 (−3.7 to −1.8)	6.3	−0.6 (−1.2 to 0.0)	7.5	1.0 (0.1 to 1.9)	51.0	−1.9 (−3.8 to −0.1)	4.3	0.2 (−0.6 to 0.9)	5.2	0.2 (−0.9 to 1.2)
Hispanic	73.6	−2.0 (−4.6 to 0.5)	5.7	−1.7 (−3.4 to 0.0)	20.8	11.9 (9.8 to 14.1)	44.6	−4.7 (−8.7 to −0.7)	4.1	0.6 (−1.0 to 2.2)	4.8	−0.5 (−2.6 to 1.6)
Other^b^	73.5	−3.5 (−4.8 to −2.1)	9.0	−0.1 (−1.0 to 0.8)	22.6	14.0 (12.7 to 15.3)	51.4	−0.9 (−3.3 to 1.5)	3.8	−0.5 (−1.4 to 0.5)	6.5	0.7 (−0.7 to 2.1)
Dual eligible												
No	80.3	[Reference]	8.4	[Reference]	8.3	[Reference]	54.9	[Reference]	4.4	[Reference]	4.7	[Reference]
Yes	74.6	−3.9 (−4.6 to −3.2)	6.8	−1.1 (−1.6 to −0.7)	12.6	1.1 (0.5 to 1.8)	49.1	−4.2 (−5.6 to −2.8)	3.1	−1.1 (−1.6 to −0.5)	4.7	−0.3 (−1.1 to 0.4)

Abbreviations: NCI, National Cancer Institute; NSCLC, non–small cell lung cancer.

^a^ Models were also adjusted for year and quarter, age, sex, urban or rural residence, area median income, area education level, Charlson Comorbidity score. Colorectal models were also adjusted for tumor location.

^b^ Includes Pacific Islander, American Indian or Alaska Native, other, and unknown.

In sensitivity analyses excluding IHC claims (eTable 9 in Supplement 1), molecular testing rates were similar across practice types except for at other academic centers, where rates were 6.7 (95% CI, 2.8 to 10.6) percentage points lower compared with NCI-designated cancer centers. When examining molecular testing rates among patients who received pemetrexed or bevacizumab (ie, patients likely to have nonsquamous NSCLC), rates were statistically lower at small independent practices compared with NCI-designated cancer centers (−2.5 percentage points; 95% CI, −4.3 to −0.6 percentage points) and 5.4 (95% CI, 2.4 to 8.5) percentage points lower among Asian patients (eTable 10 in Supplement 1).

Among patients with CRC, 56.9% of patients had claims for any molecular test. In adjusted analyses, compared with NCI-designated cancer centers, molecular testing rates were 3.8 (95% CI, 1.2-6.4) percentage points lower at nonacademic hospital-owned practices, 3.3 (95% CI, 0.4-6.1) percentage points lower at large independent practices, and 12.2 (95% CI, 9.1-15.3) percentage points lower at small independent practices. Use of multigene panels was also lower at hospital-owned and independent oncology practices compared with NCI-designated cancer centers. Compared with non-Hispanic White patients, testing rates were 1.9 (95% CI, 0.1-3.8) percentage points lower among Black patients, 4.7 (0.7-8.7) percentage points lower among Hispanic patients, and 5.6 (95% CI, 2.2-8.9) percentage points lower among Asian patients. These differences were not evident for multigene panels or in the sensitivity analysis excluding IHC claims (eTable 11 in Supplement 1). Dual-eligible patients had lower rates of all types of molecular testing compared with non–dual-eligible patients.

### Targeted Therapy Use

Targeted therapy use decreased over time among patients with NSCLC, from 417 of 3323 (12.5%) in 2015 to 105 of 1246 (8.4%) in 2019 (Figure 2C). Notably, erlotinib use declined from 315 patients (9.5%) to 10 patients (0.8%) while use of other targeted therapies increased from 102 patients (3.0%) to 95 patients (7.6%) (eFigure in Supplement 1). First-line immunotherapy use, either as monotherapy or in combination with chemotherapy, increased from 143 patients (4.3%) to 660 patients (53.0%). Table 2 and eTable 8 in Supplement 1 show unadjusted rates of targeted therapy use and adjusted differences. Overall, 9.1% of patients with NSCLC received targeted therapies. In adjusted analyses, targeted therapy use was approximately 6 percentage points lower at all other practice types compared with NCI-designated cancer centers. Adjusted rates of targeted therapy use were substantially higher among Asian and Hispanic vs non-Hispanic White patients, which was expected given the higher rates of *EGFR* variants in these populations.^33,34^ These findings were unchanged in a sensitivity analysis excluding diagnoses before 2017 (eTable 12 in Supplement 1).

Among patients with CRC, the rate of first-line targeted therapy use (mostly panitumumab or cetuximab) was 1074 of 22 772 (4.7%), and rates did not substantially change from 2015 through 2019 (Figure 2D). First-line immunotherapy increased from 7 of 1395 patients (0.5%) in 2015 to 79 of 1394 patients (4.9%) in 2019.

### Variation Across Practices

**Figure 3.**
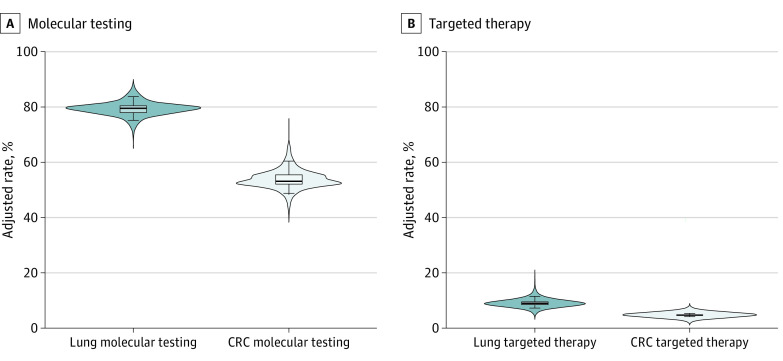
Probability Density Plots Showing Variation in Adjusted Rates for Molecular Testing and First-line Targeted Therapy Use by Practice The superimposed box and whisker plots display the median (center line), interquartile range (edges of boxes), and the 5th and 95th percentiles (whiskers) of adjusted rates. CRC indicates colorectal cancer.

Practice-level variation in molecular testing and targeted therapy use after adjustment for patient characteristics (but not practice type) are shown in Figure 3 and eTable 13 in Supplement 1. The median projected practice-level molecular testing rate for NSCLC was 79.7% (IQR, 78.0%-80.4%; 5th to 95th percentiles, 75.2-83.7%). For CRC, the median projected practice-level molecular testing rate was 53.0% (IQR, 52.1%-55.5%; 5th to 95th percentiles, 48.8%-60.5%). For targeted therapy use for NSCLC, the median adjusted practice level rate was 9.0% (IQR, 8.5%-9.4%; 5th to 95th percentiles, 7.3%-11.6%). For CRC, the median adjusted rate was 4.7% (IQR, 4.6%-4.7%; 5th to 95th percentiles, 4.3%-5.3%).

## Discussion

This study found increasing use of molecular testing for Medicare patients with metastatic NSCLC and CRC between 2015 and 2019, although rates remained low compared with recommendations that all patients receive testing. Targeted therapy use was lower than expected among patients with NSCLC, and targeted therapy use did not change among patients with metastatic CRC. Across different practice types, there was substantial variation in the use of multigene panels and targeted therapies among patients with NSCLC and some variation in molecular testing use among patients with CRC.

Overall higher molecular testing rates among patients with NSCLC compared with CRC reflect the clearer therapeutic benefits of novel therapies in NSCLC. Approximately 20% of patients with NSCLC have targetable variants, and some of these targeted therapies can improve overall survival by years.^35^ Most of the remaining 80% of patients with NSCLC could benefit from immunotherapy.^36,37^ In contrast, there is less consensus regarding the proportion of patients who should receive first-line targeted therapies for metastatic CRC. Regimens containing cetuximab and panitumumab are associated with more modest benefits, and there remains uncertainty about when they are superior to alternatives.^38,39,40^

Nonetheless, universal molecular testing has been recommended for both metastatic CRC and nonsquamous NSCLC since 2011. The increases in molecular testing seen for both NSCLC and CRC are encouraging; however, rates for both cancers remained low. Additionally, these rates included all patients with any claim for molecular testing. They did not reflect rates of comprehensive molecular profiling, which was likely lower. For example, *EGFR* testing is rarely done by IHC, so these results suggest close to 50% of patients with NSCLC may not have received *EGFR* testing in 2019.

The decline in targeted therapy use for NSCLC was surprising, and targeted therapy use remained lower than expected. The decrease may be due to decreased erlotinib use after the FDA narrowed the indication to only patients with somatic *EGFR* variants.^32^ It is also possible that immunotherapy is being used instead of targeted therapies for some patients with targetable variants, which would be concerning as these patients derive less benefit from immunotherapy.^41^ Approximately 20% of older patients with NSCLC have targetable variants, substantially higher than the 9% of patients in this cohort who received targeted therapies.^42^ Additionally, immunotherapy is recommended for nearly all patients with NSCLC without targetable variants, but in 2019, 39% of patients in this cohort received first-line regimens containing only chemotherapy. These numbers suggest many patients did not receive the most efficacious first-line therapies.

Dual-eligible status and practice type were most associated with molecular testing rates. Dual eligibility was associated with lower use of all types of molecular testing among patients with NSCLC and CRC, consistent with prior research showing that insurance type is associated with use of molecular testing.^13,17^ Molecular testing rates among patients with NSCLC were similar across practice types, but use of multigene panels was lower at all practice types compared with NCI-designated cancer centers. For CRC, molecular testing rates were lower at nonacademic oncology practices, especially small independent practices. The lower testing rates for Black patients with NSCLC and Black, Asian, and Hispanic patients with CRC were consistent with prior work showing racial disparities in access to recommended oncology care.^14^

The lower targeted therapy use for patients with NSCLC at all practice types compared with NCI-designated cancer centers could reflect referral bias; patients with targetable variants may be more likely to seek care at NCI-designated cancer centers. However, the rate of targeted therapy use at NCI-designated cancer centers more closely aligns with the expectation that approximately 20% of patients with NSCLC have targetable variants, suggesting the lower rates at other practice types may reflect underuse of targeted therapies. These differences could be related to more comprehensive molecular profiling for patients at NCI-designated cancer centers, as suggested by greater use of multigene panels at NCI-designated cancer centers.

Overall, we observed moderate variation in molecular testing across practice type after adjusting for patient characteristics, with more variation in CRC. The variation in targeted therapy use across practices was relatively small. However, these differences may be consequential in NSCLC, where the 4.3–percentage point difference between the 5th and 95th percentiles represents an approximate 20% relative difference in treatment of targetable variants.

### Limitations

This analysis has several limitations. We studied older adults enrolled in fee-for-service Medicare, so the generalizability of our findings requires further study. We lacked information about diagnosis dates and staging to identify patients with newly diagnosed metastatic carcinomas. For the NSCLC cohort, the absence of information about histology in claims data prevented us from identifying a cohort of patients with adenocarcinomas, those most likely to have targetable variants. However, our selection criteria were specific to treatment for metastatic carcinomas and these results are consistent with prior studies using data with diagnosis, histology and staging details.^12,14,15,16,43^ By selecting patients based on treatments, we excluded patients who never received treatment, which likely represent a substantial proportion of Medicare patients with metastatic cancer; therefore, our findings likely overestimate use of testing among all patients with newly diagnosed cancer.

For some procedure codes, particularly for IHC codes, we could not distinguish between diagnostic and predictive biomarker testing (ie, IHC staining for TTF-1 or p40 vs IHC staining for PD-L1 or *ALK* in NSCLC) or identify instances where pathologists billed for codes different from the assays performed. We also did not capture testing and treatments that were not billed to Medicare (eg, testing conducted at academic centers and billed to private insurance or treatments received through pharmaceutical assistance programs), which could have led to underestimates of testing and treatment. Additionally, the introduction of new procedure codes during the study period may have affected the rates of some claims, particularly multigene assays.^44^ However, the observed trends and model results were consistent across multiple sensitivity analyses. Notably, unbilled testing is likely more common at NCI-designated cancer centers, where we saw the highest testing rates; thus, unbilled testing at these centers may have underestimated differences across practice types.

## Conclusion

The findings of this study suggest that there remains substantial underuse of molecular testing and targeted therapies, with variation by practice type and patient characteristics. The patterns observed here suggest that the practice where a patient is treated may impact access to recommended testing and treatments and that socioeconomic disparities in access to testing and treatment persist. Efforts to improve access to molecular testing and targeted therapies are important to ensure all patients benefit from advances in oncology care.

## Notes

Supplement 1. **eTable 1.** Systemic Therapies for Non–Small Cell Lung Cancer **eTable 2.** Systemic Therapies for Colorectal Cancer **eTable 3.** Billing Codes for Lung Cancer Resection Surgeries **eTable 4.** Billing Codes for Radiation Therapy **eTable 5.** Billing Codes for Colorectal Cancer Resection Surgeries **eTable 6.** Billing Codes for Molecular Tests **eTable 7.** *ICD-10* Codes for Colorectal Cancer Categorized by Site **eTable 8.** Full Regression Results **eTable 9.** NSCLC Molecular Testing Sensitivity Analysis (Excluding IHC) **eTable 10.** NSCLC Molecular Testing Sensitivity Analysis (Bevacizumab/Pemetrexed) **eTable 11.** CRC Molecular Testing Sensitivity Analysis (Excluding IHC) **eTable 12.** NSCLC Targeted Therapy Sensitivity Analysis (2017-2019) **eTable 13.** Variation Across Oncology Practices **eFigure.** Trends in Use of Erlotinib and Other Targeted Therapies

Supplement 2.
Data Sharing Statement
