# Supplementary material for: Practice-Level Variation in Molecular Testing and Use of Targeted Therapy for Patients With Non–Small Cell Lung Cancer and Colorectal Cancer
Source: JAMA Netw Open. 2023 Apr 28;6(4):e2310809. doi: 10.1001/jamanetworkopen.2023.10809 (PMC10148196; doi:10.1001/jamanetworkopen.2023.10809)
Supplement: Supplement 1. — eTable 1. Systemic Therapies for Non–Small Cell Lung Cancer eTable 2. Systemic Therapies for Colorectal Cancer eTable 3. Billing Codes for Lung Cancer Resection Surgeries eTable 4. Billing Codes for Radiation Therapy eTable 5. Billing Codes for Colorectal Cancer Resection Surgeries eTable 6. Billing Codes for Molecular Tests eTable 7. ICD-10 Codes for Colorectal Cancer Categorized by Site eTable 8. Full Regression Results eTable 9. NSCLC Molecular Testing Sensitivity Analysis (Excluding IHC) eTable 10. NSCLC Molecular Testing Sensitivity Analysis (Bevacizumab/Pemetrexed) eTable 11. CRC Molecular Testing Sensitivity Analysis (Excluding IHC) eTable 12. NSCLC Targeted Therapy Sensitivity Analysis (2017-2019) eTable 13. Variation Across Oncology Practices eFigure. Trends in Use of Erlotinib and Other Targeted Therapies [file jamanetwopen-e2310809-s001.pdf]

Supplemental Online Content

Roberts TJ, Kehl KL, Brooks GA, et al. Practice-level variation in molecular testing and use of targeted therapy for patients with non–small cell lung cancer and colorectal cancer. *JAMA Netw Open.* 2023;6(4):e2310809.
doi:10.1001/jamanetworkopen.2023.10809

eTable 1. Systemic Therapies for Non–Small Cell Lung Cancer

eTable 2. Systemic Therapies for Colorectal Cancer

eTable 3. Billing Codes for Lung Cancer Resection Surgeries

eTable 4. Billing Codes for Radiation Therapy

eTable 5. Billing Codes for Colorectal Cancer Resection Surgeries

eTable 6. Billing Codes for Molecular Tests

eTable 7. *ICD-10* Codes for Colorectal Cancer Categorized by Site

eTable 8. Full Regression Results

eTable 9. NSCLC Molecular Testing Sensitivity Analysis (Excluding IHC)

eTable 10. NSCLC Molecular Testing Sensitivity Analysis (Bevacizumab/Pemetrexed)

eTable 11. CRC Molecular Testing Sensitivity Analysis (Excluding IHC)

eTable 12. NSCLC Targeted Therapy Sensitivity Analysis (2017-2019)

eTable 13. Variation Across Oncology Practices

eFigure. Trends in Use of Erlotinib and Other Targeted Therapies

This supplemental material has been provided by the authors to give readers additional information about their work.

© 2023 Roberts TJ et al. *JAMA Network Open.*

eTable 1. Systemic Therapies for Non–Small Cell Lung Cancer

Therapeutic class	Drug name	Part B or D	HCPCS Codes	NDC Codes
**Targeted Therapies**	Osimertinib	Part D	NA	00310-1349-30<br>00310-1350-30
	Erlotinib	Part D	NA	72485-0217-30<br>72485-0219-30<br>72485-0218-30<br>00378-7132-93<br>00378-7133-93<br>00378-7131-93<br>63304-0135-30<br>63304-0096-30<br>63304-0095-30<br>00093-7664-56<br>00093-7663-56<br>54569-5848-00<br>54569-5847-00<br>50242-0062-01<br>50242-0064-01<br>50242-0063-01<br>54868-5290-00<br>54868-5447-00<br>54868-5474-00
	Afatinib	Part D	NA	00597-0141-30<br>00597-0137-30<br>00597-0138-30
	Gefitinib	Part D	J8565	00310-0482-30
	Alectinib	Part D	NA	50242-0130-01
	Brigatinib	Part D	NA	76189-0113-21<br>76189-0113-18<br>63020-0180-30<br>63020-0090-30<br>63020-0113-30<br>63020-0090-07<br>63020-0198-30
	Ceritinib	Part D	NA	00078-0694-84<br>00078-0640-70

© 2023 Roberts TJ et al. *JAMA Network Open.*

	Crizotinib	Part D	NA	00069-8140-20<br>00069-8141-20
	Lorlatinib	Part D	NA	00069-0231-01<br>00069-0227-01
	Dabrafenib	Part D	NA	00173-0847-08<br>00078-0682-66<br>00173-0846-08<br>00078-0681-66
	Trametinib	Part D	NA	00078-0668-15<br>00173-0849-13<br>00078-0666-15<br>00173-0848-13
	Vemurafenib	Part D	NA	50242-0090-02<br>50242-0090-01
	Dacomitinib	Part D	NA	00069-0197-30<br>00069-2299-30<br>00069-1198-30
	Entrectinib	Part D	NA	50242-0091-30<br>50242-0094-90
	Larotrectinib	Part D	NA	50419-0391-01<br>50419-0392-01<br>71777-0391-01<br>71777-0392-01<br>71777-0390-01
**Immunotherapies**	Pembrolizumab	Part B	C9027; J9271	00006-3026-02<br>00006-3026-01<br>00006-3029-01<br>00006-3029-02<br>00006-3026-04
	Durvalumab	Part B	J9173; C9492	00310-4611-50<br>00310-4500-12
	Atezolizumab	Part B	J9022; C9483	50242-0918-01<br>50242-0917-01
	Ipilimumab	Part B	J9228; C9284	00003-2328-22<br>00003-2327-11
	Nivolumab	Part B	C9453; J9299	00003-3774-12<br>00003-3734-13<br>00003-3772-11

© 2023 Roberts TJ et al. *JAMA Network Open.*

**Chemotherapy and other systemic therapies**	Carboplatin	Part B	J9045	55390-0221-01<br>55390-0222-01<br>55390-0220-01<br>61703-0339-62<br>61703-0339-61<br>61703-0339-63<br>16729-0295-31<br>16729-0295-12<br>16729-0295-33<br>16729-0295-34<br>47781-0606-94<br>47781-0604-27<br>47781-0603-20<br>47781-0605-94<br>63323-0167-20<br>63323-0166-10<br>63323-0168-00<br>10019-0912-03<br>10019-0912-02<br>10019-0916-01<br>10019-0917-01<br>10019-0912-01<br>10019-0915-01<br>55390-0151-01<br>55390-0152-01<br>55390-0150-01<br>55390-0153-01<br>55390-0155-01<br>55390-0154-01<br>55390-0156-01<br>63323-0172-45<br>63323-0172-60<br>63323-0167-21<br>63323-0172-15<br>63323-0172-05<br>10139-0060-15<br>10139-0060-45<br>10139-0060-05

© 2023 Roberts TJ et al. *JAMA Network Open.*

				00409-1129-12	
				00409-1129-11	
				00409-1129-10	
				61703-0339-56	
				61703-0339-22	
				61703-0339-50	
				61703-0339-18	
				50742-0445-05	
				50742-0447-45	
				50742-0448-60	
				50742-0446-15	
				71288-0100-15	
				71288-0100-05	
				71288-0100-45	
				71288-0100-51	
				67457-0493-46	
				67457-0494-61	
				67457-0492-15	
				67457-0491-54	
				67817-0067-12	
				67817-0063-12	
				67817-0066-12	
				67817-0061-12	
				15210-0067-12	
				15210-0066-12	
				15210-0063-12	
				15210-0061-12	
				50111-0966-76	
				50111-0965-76	
				25021-0202-51	
				25021-0202-15	
				25021-0202-05	
				25021-0202-45	
				66758-0047-01	
				66758-0047-04	
				66758-0047-02	
				66758-0047-03	
				47335-0300-40	

© 2023 Roberts TJ et al. *JAMA Network Open.*

				47335-0284-40
				47335-0151-40
				47335-0150-40
				00703-4246-01
				00703-3264-01
				00703-3246-11
				00703-3266-01
				00703-3244-11
				00703-3249-11
				00703-3248-11
				00703-3268-71
				00703-4244-01
				00703-4248-01
				00703-3268-01
				00703-3278-01
				00703-3276-01
				00703-3274-01
				00703-4239-01
				50111-0967-76
				00591-3687-11
				00591-2219-11
				00591-2220-11
				00591-3337-12
				00591-3454-60
				00591-3336-26
				00591-3338-89
				67457-0608-20
				61703-0360-22
				61703-0360-18
				61703-0360-50
				00703-4248-91
				00015-3230-11
				00015-3232-11
				00015-3233-11
				00015-3231-11
				00015-3210-30
				00015-3216-30
				00015-3211-76

© 2023 Roberts TJ et al. *JAMA Network Open.*

				00015-3214-30<br>00015-3213-30<br>00015-3212-30<br>00015-3211-30<br>00015-3215-30<br>00015-3212-76<br>00015-3210-76<br>00015-3214-29<br>00015-3213-29<br>00015-3215-29<br>00703-4239-81<br>00703-4248-81<br>00703-4246-81<br>00703-4244-81
	Cisplatin	Part B	C9418; J9060; J9062	16729-0288-11<br>16729-0288-38<br>47781-0610-23<br>47781-0609-25<br>70860-0206-50<br>70860-0206-51<br>10019-0910-01<br>10019-0910-02<br>55390-0099-01<br>55390-0112-99<br>55390-0112-50<br>68001-0283-24<br>68001-0283-33<br>68001-0283-32<br>68001-0283-27<br>51552-1076-09<br>63323-0103-51<br>63323-0103-64<br>63323-0103-65<br>00143-9505-01<br>00143-9504-01<br>62991-2849-01<br>62991-2849-02<br>67457-0425-51

© 2023 Roberts TJ et al. *JAMA Network Open.*

				00069-0084-07<br>67457-0424-10<br>00069-0081-01<br>49452-2078-02<br>49452-2078-01<br>49452-2078-03<br>00703-5747-11<br>00703-5748-11<br>44567-0511-01<br>44567-0510-01<br>44567-0530-01<br>44567-0509-01<br>63323-0103-91<br>63323-0103-95<br>55390-0414-99<br>55390-0187-01<br>55390-0414-50<br>00015-3070-20<br>00015-3072-20<br>00015-3221-22<br>00015-3220-22<br>00015-3220-26<br>00015-3221-26
	Ramucirumab	Part B	C9025; J9308	00002-7678-01<br>00002-7669-01
	Vinorelbine	Part B	C9440; J9390	55390-0267-01<br>55390-0268-01<br>64370-0250-01<br>64370-0210-01<br>00173-0656-44<br>00173-0656-01<br>60831-3086-02<br>64370-0532-02<br>64370-0532-01<br>64370-3086-02<br>64370-3086-01<br>60831-3086-01<br>67457-0479-53

© 2023 Roberts TJ et al. *JAMA Network Open.*

				00069-0099-01<br>00069-0103-03<br>67457-0431-11<br>25021-0204-01<br>25021-0204-05<br>66758-0045-01<br>66758-0045-02<br>45963-0607-55<br>45963-0607-56<br>00069-0205-50<br>00069-0205-10<br>67457-0481-01<br>10019-0970-02<br>10019-0970-01<br>59911-5958-01<br>59911-5959-01<br>63323-0148-05<br>63323-0148-01<br>61703-0341-06<br>61703-0341-09<br>00703-4183-01<br>00703-4183-91<br>00703-4183-81<br>00703-4182-81<br>00703-4182-91<br>00703-4182-01<br>55390-0069-01<br>55390-0070-01
	Etoposide	Part B	J8560; J9181; J9182; WW030; WW031; WW032; C9425; C9414	54569-5718-00<br>00703-5667-01<br>00074-1485-02<br>00074-1485-01<br>00074-1485-03<br>00703-5646-01<br>00703-5643-01<br>16729-0114-11<br>16729-0114-31<br>16729-0114-08

© 2023 Roberts TJ et al. *JAMA Network Open.*

				00186-1571-31
				10019-0930-02
				10019-0930-01
				68001-0265-24
				68001-0265-22
				68001-0265-27
				68001-0265-23
				68001-0265-26
				68001-0265-25
				63323-0104-25
				63323-0104-05
				63323-0104-50
				00703-5668-01
				00143-9510-01
				00143-9512-01
				00143-9511-01
				00209-3080-20
				00209-3090-20
				00209-3060-22
				00209-3070-20
				38779-0784-03
				38779-0784-06
				00378-3266-94
				54868-5355-01
				54868-5355-00
				54868-5355-02
				51927-2772-00
				00364-3028-53
				58406-0714-18
				58406-0711-12
				51079-0965-05
				55390-0291-01
				55390-0293-01
				55390-0292-01
				16729-0262-31
				55390-0492-01
				55390-0491-01
				55390-0493-01

© 2023 Roberts TJ et al. *JAMA Network Open.*

				00013-7356-88<br>00013-7346-94<br>00013-7336-91<br>00703-5653-01<br>00703-5656-01<br>00703-5657-01<br>00703-5657-91<br>00703-5656-91<br>00015-3084-20<br>00015-3061-20<br>00015-3062-20<br>00015-3091-45<br>00015-3095-20<br>00015-3062-24<br>00015-3095-30<br>00015-3061-24<br>00015-3404-20
	Gemcitabine	Part B	J9201	16729-0391-30<br>16729-0426-05<br>68001-0342-34<br>68001-0348-36<br>16729-0117-11<br>16729-0092-03<br>16729-0118-38<br>16729-0423-33<br>16729-0419-03<br>60505-6113-06<br>60505-6114-00<br>60505-6115-02<br>63323-0125-50<br>70860-0205-50<br>55390-0391-50<br>55390-0391-10<br>68001-0282-26<br>68001-0282-27<br>68001-0282-25<br>55111-0686-07

				55111-0687-25<br>63323-0102-13<br>63323-0125-53<br>63323-0102-10<br>63323-0126-00<br>23155-0213-31<br>23155-0214-31<br>00409-0187-01<br>00409-0185-01<br>00409-0183-01<br>00409-0186-01<br>00409-0182-01<br>00409-0181-01<br>71288-0114-50<br>71288-0113-10<br>67457-0616-10<br>00069-3858-10<br>67457-0462-01<br>67457-0617-30<br>67457-0618-10<br>16714-0909-01<br>16714-0930-01<br>25021-0209-50<br>25021-0208-10<br>25021-0239-26<br>25021-0239-05<br>25021-0239-52<br>47335-0154-40<br>47335-0153-40<br>00703-5775-01<br>00703-5778-01<br>45963-0619-59<br>45963-0612-57<br>45963-0623-57<br>45963-0636-60<br>45963-0624-58<br>00591-3563-55<br>00591-3562-79

© 2023 Roberts TJ et al. *JAMA Network Open.*

				00069-3857-10<br>67457-0464-20<br>67457-0463-02<br>00069-3859-10<br>25021-0234-10<br>25021-0235-50<br>00781-3282-75<br>00781-3283-79<br>45963-0620-60<br>23155-0528-31<br>23155-0529-31<br>00409-0181-25<br>00409-0183-25<br>00409-0182-25<br>00002-7501-01<br>00002-7502-01<br>62756-0974-60<br>62756-0219-60<br>62756-0102-60<br>62756-0073-60<br>62756-0746-60<br>62756-0008-60<br>62756-0614-60<br>62756-0321-60<br>62756-0438-60<br>63323-0125-94<br>63323-0102-94<br>23155-0484-31<br>23155-0483-31<br>62756-0533-60
	Pemetrexed	Part B	J9305; C9213	00002-7640-01<br>00002-7623-01
	Paclitaxel	Part B	J9265; J9267; C9431	00172-3756-95<br>00172-3753-96<br>00172-3754-94<br>00172-3756-75<br>00172-3753-77<br>00172-3754-73

© 2023 Roberts TJ et al. *JAMA Network Open.*

				47781-0594-07
				47781-0593-07
				47781-0595-07
				10518-0102-09
				10518-0102-08
				10518-0102-07
				70860-0200-50
				70860-0200-05
				70860-0200-17
				51991-0936-98
				51991-0937-98
				51991-0938-98
				63323-0763-17
				63323-0763-06
				63323-0763-52
				63323-0763-05
				63323-0763-50
				63323-0763-16
				61703-0342-50
				61703-0342-22
				61703-0342-09
				00074-4335-01
				00074-4335-02
				00074-4335-04
				00069-0078-01
				00069-0076-01
				00069-0079-01
				67457-0471-52
				67457-0434-51
				67457-0449-17
				25021-0213-17
				25021-0213-50
				25021-0213-05
				66758-0043-03
				66758-0043-02
				66758-0043-01
				45963-0613-56
				45963-0613-53

© 2023 Roberts TJ et al. *JAMA Network Open.*

				00555-1985-14
				00555-1984-14
				45963-0613-59
				00703-4768-01
				00703-4766-01
				00703-4767-01
				00703-4764-01
				51079-0963-01
				51079-0962-01
				51079-0961-01
				55390-0114-05
				55390-0114-20
				55390-0114-50
				44567-0505-01
				44567-0506-01
				55390-0314-50
				55390-0314-05
				55390-0314-20
				55390-0304-05
				55390-0304-50
				55390-0304-20
				00172-3755-31
				55390-0514-20
				55390-0514-05
				55390-0514-50
				00703-4764-81
				00703-4766-81
				00703-4768-81
				45963-0613-83
				45963-0613-89
				45963-0613-86
				00015-3475-30
				00015-3479-11
				00015-3476-30
				00015-3456-99
				00015-3475-20
				00015-3476-20

	Albumin-bound paclitaxel	Part B	J9264; C9127	68817-0134-50
	Bevacizumab	Part B	J9035	71266-8005-02<br>71266-8005-01<br>50242-0061-01<br>50242-0060-10<br>50242-0060-02<br>50242-0060-01<br>50242-0061-10
	Docetaxel	Part B	J9170; J9171	47335-0285-41<br>47335-0286-41<br>16729-0267-64<br>16729-0267-63<br>16729-0267-65<br>16729-0231-65<br>16729-0231-63<br>16729-0120-50<br>16729-0120-49<br>16729-0231-64<br>45963-0734-74<br>60505-6037-06<br>60505-6035-06<br>43066-0010-01<br>43066-0006-01<br>43066-0001-01<br>43598-0259-40<br>43598-0258-11<br>42367-0121-25<br>42367-0121-29<br>42367-0121-21<br>00409-0367-01<br>00409-0201-02<br>00409-0201-20<br>00409-0368-01<br>00409-0366-01<br>00409-0201-10<br>50742-0428-02<br>50742-0463-16

© 2023 Roberts TJ et al. *JAMA Network Open.*

				50742-0431-08	
				63739-0971-17	
				63739-0932-11	
				67457-0531-02	
				67457-0781-08	
				67457-0532-08	
				67457-0533-16	
				16714-0500-01	
				16714-0465-01	
				00069-9141-22	
				00069-9142-22	
				00069-9144-11	
				00069-9142-11	
				00069-9141-11	
				25021-0222-01	
				25021-0222-07	
				25021-0222-04	
				25021-0245-04	
				25021-0245-01	
				66758-0050-03	
				66758-0050-01	
				66758-0050-02	
				00703-5720-01	
				00703-5730-01	
				45963-0734-54	
				45963-0790-56	
				45963-0765-52	
				45963-0734-52	
				00955-1022-08	
				00955-1020-01	
				00955-1021-04	
				39822-2180-01	
				39822-2120-01	
				39822-2200-01	
				43598-0611-11	
				43598-0610-40	
				00409-0201-26	
				00409-0201-27	

© 2023 Roberts TJ et al. *JAMA Network Open.*

				00409-0201-25<br>66758-0950-02<br>66758-0950-04<br>66758-0950-03<br>00075-8001-20<br>00075-8004-04<br>00075-8003-01<br>00075-8001-80
	Cabozantinib	Part D	NA	42388-0024-26<br>42388-0025-26<br>42388-0023-26<br>42388-0013-14<br>42388-0011-14<br>42388-0012-14
	Vandetanib	Part D	NA	58468-7820-03<br>58468-7840-03<br>00310-7840-30<br>00310-7820-30<br>00310-7810-30<br>00310-7830-30
	Bevacizumab-awwb	Part B	Q5107	55513-0207-01<br>55513-0206-01

© 2023 Roberts TJ et al. *JAMA Network Open.*

eTable 2. Systemic Therapies for Colorectal Cancer

Therapeutic class	Drug name	Part B or D	HCPCS Code(s)	NDC Codes
**Targeted therapies**	Cetuximab	Part B	J9055	66733-0958-23<br>66733-0948-23
	Panitumumab	Part B	J9303; C9235	55513-0954-01<br>55513-0956-01<br>55513-0955-01
	Dabrafenib	Part D	NA	00173-0847-08<br>00078-0682-66<br>00173-0846-08<br>00078-0681-66
	Trametinib	Part D	NA	00078-0668-15<br>00173-0849-13<br>00078-0666-15<br>00173-0848-13
	Vemurafenib	Part D	NA	50242-0090-02<br>50242-0090-01
**Immunotherapies**	Pembrolizumab	Part B	C9027; J9271	00006-3026-02<br>00006-3026-01<br>00006-3029-01<br>00006-3029-02<br>00006-3026-04
	Ipilimumab	Part B	J9228; C9284	00003-2328-22<br>00003-2327-11
	Nivolumab	Part B	C9453; J9299	00003-3774-12<br>00003-3734-13<br>00003-3772-11
**Chemotherapy and other systemic therapies**	Oxaliplatin	Part B	J9263; C9205	00024-0592-40<br>00024-0596-02<br>00024-0597-04<br>00024-0590-10<br>00024-0591-20<br>16729-0332-05<br>16729-0332-03<br>60505-6132-07<br>60505-6132-06<br>70860-0201-10<br>70860-0201-20

© 2023 Roberts TJ et al. *JAMA Network Open.*

				43066-0018-01
				43066-0014-01
				68001-0341-29
				68001-0341-28
				68001-0341-37
				68001-0341-36
				51991-0923-98
				51991-0922-98
				51991-0219-98
				51991-0218-98
				63323-0175-30
				63323-0176-50
				63323-0650-17
				63323-0650-27
				63323-0650-20
				63323-0650-10
				63323-0750-20
				63323-0750-10
				63323-0211-10
				63323-0212-20
				61703-0363-18
				61703-0363-40
				61703-0363-22
				50742-0405-10
				50742-0406-20
				71288-0101-20
				71288-0101-10
				00069-1010-01
				00069-0074-01
				67457-0442-20
				67457-0469-10
				00069-0070-01
				00069-0067-01
				67457-0476-10
				72603-0301-01
				16714-0728-01
				16714-0727-01
				72603-0101-01

© 2023 Roberts TJ et al. *JAMA Network Open.*

				25021-0212-50<br>25021-0233-10<br>25021-0233-20<br>25021-0211-20<br>00781-3315-70<br>00781-3317-80<br>66758-0053-02<br>66758-0053-01<br>47335-0046-40<br>47335-0178-40<br>41616-0176-40<br>47335-0176-40<br>41616-0178-40<br>47335-0047-40<br>45963-0637-49<br>45963-0638-58<br>45963-0611-53<br>00703-3985-01<br>00703-3986-01<br>45963-0611-59<br>00955-1725-10<br>00955-1727-20<br>00781-9317-80<br>00781-9315-70<br>00955-1731-10<br>00955-1733-20
	5-FU	Part B	J9190	63370-0095-35<br>63370-0095-15<br>63370-0095-25<br>49452-3175-04<br>49452-3175-01<br>49452-3175-03<br>49452-3175-05<br>49452-3175-02<br>00013-1046-94<br>00013-1036-91<br>00013-1056-94<br>00703-3019-11

© 2023 Roberts TJ et al. *JAMA Network Open.*

				00703-3018-12
				00703-3015-13
				00703-3019-12
				00703-3015-11
				00187-5200-30
				54868-5450-00
				00066-7150-30
				54569-1100-00
				54569-1566-00
				00187-3204-47
				54868-0951-00
				54868-0951-01
				60346-0548-81
				58016-2017-01
				00187-3202-10
				00187-3202-02
				00187-3203-10
				00187-3203-02
				00187-3204-26
				00187-3600-60
				54569-1565-00
				00023-0810-30
				16110-0812-30
				00023-0812-30
				58016-9106-01
				54569-1406-00
				54569-6279-00
				16729-0276-11
				16729-0276-38
				16729-0276-67
				16729-0276-68
				16729-0276-03
				10019-0950-02
				68001-0266-30
				68001-0266-27
				68001-0266-31
				68001-0266-32
				51552-0733-05

© 2023 Roberts TJ et al. *JAMA Network Open.*

				51552-0733-01<br>51552-0733-02<br>51552-0733-04<br>63323-0117-20<br>63323-0117-10<br>63323-0117-51<br>63323-0117-61<br>00469-1710-60<br>00469-1711-00<br>00469-1710-30<br>00469-1710-40<br>10139-0063-10<br>10139-0063-20<br>51432-0409-10<br>51432-0470-10<br>00395-8080-62<br>00395-8080-19<br>62991-1486-03<br>62991-1486-01<br>62991-1486-02<br>62991-1486-04<br>51862-0362-40<br>66530-0249-40<br>61703-0409-67<br>61703-0409-53<br>61703-0409-32<br>38779-3185-06<br>38779-0025-10<br>38779-0025-01<br>38779-0025-25<br>38779-0025-06<br>38779-0025-03<br>38779-0025-09<br>38779-0025-05<br>38779-0025-04<br>38779-3185-05<br>38779-3185-09<br>38779-3185-01

© 2023 Roberts TJ et al. *JAMA Network Open.*

				38779-3185-04<br>38779-3185-03<br>10139-0063-01<br>10139-0063-11<br>10139-0063-50<br>10139-0063-12<br>00378-4791-06<br>66530-0258-30<br>68682-0004-31<br>68682-0085-31<br>21695-0829-40<br>54868-6293-00<br>51927-1085-00<br>51309-0217-50<br>51309-0217-98<br>51309-0217-20<br>25021-0215-98<br>25021-0215-99<br>66758-0044-03<br>66758-0044-01<br>43547-0259-01<br>43547-0258-01<br>39769-0012-90<br>39769-0012-10<br>39769-0012-40<br>51672-4063-01<br>51672-4118-06<br>51672-4062-01<br>00187-3953-64<br>00702-1710-30<br>63323-0117-69<br>63323-0117-19<br>63323-0117-59<br>63323-0117-58<br>63323-0117-18<br>63323-0117-28<br>63323-0117-68<br>64370-0421-40

© 2023 Roberts TJ et al. *JAMA Network Open.*

				28105-0421-40
	Capecitabine	Part B (note- oral drug- check for HCPCS codes AND check for NDCs in DME file)	J8520; J8521; WW089; WW090; WW091; WW093; WW094; WW096	16729-0072-12<br>16729-0073-29<br>60687-0149-11<br>60687-0149-94<br>65162-0844-16<br>65162-0843-06<br>72485-0204-60<br>72485-0205-12<br>67877-0458-60<br>67877-0459-12<br>59651-0205-08<br>59651-0204-60<br>42291-0167-12<br>42291-0166-60<br>42291-0190-60<br>42291-0191-12<br>72606-0554-01<br>72606-0555-01<br>69097-0949-03<br>69097-0948-08<br>51407-0095-60<br>51407-0096-12<br>00054-0271-21<br>00054-0272-23<br>51079-0510-01<br>51079-0510-05<br>00378-2512-78<br>00378-2511-91<br>16714-0467-01<br>16714-0468-01<br>72205-0006-60<br>72205-0007-92<br>64980-0277-12<br>64980-0276-06<br>62756-0239-20<br>62756-0238-86<br>00093-7474-89

© 2023 Roberts TJ et al. *JAMA Network Open.*

				00093-7473-06<br>50268-0154-11<br>50268-0154-13<br>54569-5717-00<br>00004-1100-20<br>00004-1101-50<br>00004-1101-75<br>54868-4143-03<br>54868-4143-01<br>54868-5260-04<br>54868-5260-05<br>54868-5260-06<br>54868-5260-07<br>54868-5260-08<br>54868-5260-09<br>54868-5260-00<br>54868-5260-01<br>54868-5260-03<br>54868-4143-00<br>54868-4143-02<br>54868-5260-02<br>00004-1100-51<br>00004-1101-16
	Ramucirumab	Part B	C9025; J9308	00002-7678-01<br>00002-7669-01
	Bevacizumab	Part B	J9035	71266-8005-02<br>71266-8005-01<br>50242-0061-01<br>50242-0060-10<br>50242-0060-02<br>50242-0060-01<br>50242-0061-10
	Irinotecan	Part B	J9206	61703-0349-61<br>61703-0349-62<br>00009-7529-01<br>00009-7529-02<br>00009-7529-05<br>00009-7529-04

				00009-7529-03
				00009-1111-01
				00009-1111-02
				00143-9583-01
				00703-4434-91
				18111-0002-02
				18111-0002-03
				60505-6128-00
				60505-6128-01
				10518-0103-10
				10518-0103-11
				59923-0714-02
				59923-0715-05
				59923-0702-02
				59923-0702-05
				72485-0212-05
				72485-0211-02
				55390-0295-01
				55390-0296-01
				68001-0284-34
				68001-0284-25
				63323-0193-02
				63323-0193-05
				63323-0193-52
				63323-0193-55
				59762-7529-01
				59762-7529-02
				23155-0179-32
				23155-0179-31
				00143-9701-01
				00143-9702-01
				61703-0349-09
				61703-0349-16
				61703-0349-36
				50742-0402-05
				50742-0401-02
				16714-0725-01
				16714-0726-01

© 2023 Roberts TJ et al. *JAMA Network Open.*

				25021-0230-05<br>25021-0214-05<br>25021-0214-02<br>25021-0230-02<br>25021-0200-02<br>25021-0200-05<br>66758-0048-01<br>00781-3066-72<br>00781-3066-75<br>66758-0048-02<br>00703-4432-11<br>00703-4437-11<br>00703-4434-11<br>45963-0614-55<br>45963-0614-51<br>00591-3189-02<br>00591-3189-26<br>10019-0934-02<br>10019-0934-01<br>10019-0934-79<br>10019-0934-17<br>00703-4432-81<br>45963-0614-85<br>45963-0614-81<br>00703-4434-81
	Regorafinib	Part D	NA	50419-0171-01<br>50419-0171-03
	Trifluridine/tipiracil	Part D	NA	64842-1025-01<br>64842-1025-03<br>64842-1020-02<br>64842-1020-01<br>64842-1020-03<br>64842-1025-02

eTable 3. Billing Codes for Lung Cancer Resection Surgeries

CPT Code	Definition
32440	Removal of lung, pneumonectomy
32442	Removal of lung, pneumonectomy; with resection of segment of trachea followed by broncho-tracheal anastomosis (sleeve pneumonectomy)
32445	Removal of lung, pneumonectomy; extrapleural
32480	Removal of lung, other than pneumonectomy; single lobe (lobectomy)
32482	Removal of lung, other than pneumonectomy; 2 lobes (bilobectomy)
32484	Removal of lung, other than pneumonectomy; single segment (segmentectomy)
32486	Removal of lung, other than pneumonectomy; with circumferential resection of segment of bronchus followed by broncho-bronchial anastomosis (sleeve lobectomy)
32488	Removal of lung, other than pneumonectomy; with all remaining lung following previous removal of a portion of lung (completion pneumonectomy)
32663	Thoracoscopy, surgical; with lobectomy (single lobe)
32500	Removal of lung, other than total pneumonectomy; wedge resection, single or multiple
32505	Thoracotomy; with therapeutic wedge resection (eg, mass, nodule), initial
32506	Thoracotomy; with therapeutic wedge resection (eg, mass or nodule), each additional resection, ipsilateral (List separately in addition to code for primary procedure)
32507	Thoracotomy; with diagnostic wedge resection followed by anatomic lung resection (List separately in addition to code for primary procedure)
32657	Thoracoscopy, surgical; with wedge resection of lung, single or multiple
32666	Thoracoscopy, surgical; with therapeutic wedge resection (eg, mass, nodule), initial unilateral
32667	Thoracoscopy, surgical; with therapeutic wedge resection (eg, mass or nodule), each additional resection, ipsilateral (List separately in addition to code for primary procedure)
32668	Thoracoscopy, surgical; with diagnostic wedge resection followed by anatomic lung resection (List separately in addition to code for primary procedure)
32669	thorascopy, surgical; with removal of a single lung segment (segmentectomy)
32670	Thoracoscopy, surgical; with removal of two lobes (bilobectomy)
32671	Thoracoscopy, surgical; with removal of lung (pneumonectomy)

ICD-9 Procedure Code	Definition
32.20	thorascopic excision of lesion or tissue of lung
32.28	endoscopic excision or destruction of lesion or tissue of lung
32.29	other local eceisions or destruction of lesions or tissue of lung
32.50	thorascopic pneumonectomy
32.59	other and unspecified pneumonectomy
32.41	Thoracoscopic lobectomy of lung
32.49	Other lobectomy of lung
32.30	Thoracoscopic segmental resection of lung
32.39	Other and unspecified segmental resection of lung
**ICD-10 Procedure Code**	**Definition**
0BTK4ZZ	Resection of Right Lung, Percutaneous Endoscopic Approach
0BTL4ZZ	Resection of Left Lung, Percutaneous Endoscopic Approach
0BTM4ZZ	Resection of Bilateral Lungs, Percutaneous Endoscopic Approach
0BTK0ZZ	Resection of Right Lung, Open Approach
0BTL0ZZ	Resection of Left Lung, Open Approach
0BTM0ZZ	Resection of Bilateral Lungs, Open Approach
0BTC4ZZ	Resection of Right Upper Lung Lobe, Percutaneous Endoscopic Approach
0BTD4ZZ	Resection of Right Middle Lung Lobe, Percutaneous Endoscopic Approach
0BTF4ZZ	Resection of Right Lower Lung Lobe, Percutaneous Endoscopic Approach
0BTG4ZZ	Resection of Left Upper Lung Lobe, Percutaneous Endoscopic Approach
0BTJ4ZZ	Resection of Left Lower Lung Lobe, Percutaneous Endoscopic Approach
0BTC0ZZ	Resection of Right Upper Lung Lobe, Open Approach
0BTD0ZZ	Resection of Right Middle Lung Lobe, Open Approach
0BTF0ZZ	Resection of Right Lower Lung Lobe, Open Approach
0BTG0ZZ	Resection of Left Upper Lung Lobe, Open Approach
0BTJ0ZZ	Resection of Left Lower Lung Lobe, Open Approach
0BBC4ZZ	Excision of Right Upper Lung Lobe, Percutaneous Endoscopic Approach
0BBC0ZZ	Excision of right upper lung, open approach
0BBD4ZZ	Excision of Right Middle Lung Lobe, Percutaneous Endoscopic Approach
0BBD0ZZ	Excision of right middle lung lobe, open approach
0BBF4ZZ	Excision of Right Lower Lung Lobe, Percutaneous Endoscopic Approach
0BBF0ZZ	Excision of right lower lung lobe, open approach
0BBG4ZZ	Excision of Left Upper Lung Lobe, Percutaneous Endoscopic Approach
0BBG0ZZ	Excision of left upper lung lobe, open approach
0BBH4ZZ	Excision of Lung Lingula, Percutaneous Endoscopic Approach
0BBH0ZZ	Excision of lung lingula, open approach

0BBJ4ZZ	Excision of Left Lower Lung Lobe, Percutaneous Endoscopic Approach
0BBJ0ZZ	Excision of left lower lung lobe, open approach
0BBK4ZZ	Excision of Right Lung, Percutaneous Endoscopic Approach
0BBL4ZZ	Excision of Left Lung, Percutaneous Endoscopic Approach
0BTH4ZZ	Resection of Lung Lingula, Percutaneous Endoscopic Approach
0BTH0ZZ	Resection of lung lingula, open approach
0BBK0ZZ	Excision of Right Lung, Open Approach
0BBK3ZZ	Excision of Right Lung, Percutaneous Approach
0BBL0ZZ	Excision of Left Lung, Open Approach
0BBM0ZZ	Excision of Bilateral Lungs, Open Approach
0BBM4ZZ	Excision of Bilateral Lungs, Perc Endo Approach

© 2023 Roberts TJ et al. *JAMA Network Open.*

eTable 4. Billing Codes for Radiation Therapy

Radiation Type	Code Type	Code	Description
EBRT	CPT	77401	Radiation treatment delivery, superficial and/or ortho voltage, per day
EBRT	CPT	77402	Radiation treatment delivery,=>1 MeV; simple
EBRT	CPT	77403	Radiation treatment delivery, single treatment area, single port or parallel opposed ports, simple blocks or no blocks; 6-10 MeV
EBRT	CPT	77404	Radiation treatment delivery, single treatment area, single port or parallel opposed ports, simple blocks or no blocks; 11-19 MeV
EBRT	CPT	77406	Radiation treatment delivery, single treatment area, single port or parallel opposed ports, simple blocks or no blocks; 20 MeV or greater
EBRT	CPT	77407	Radiation treatment delivery, =>1 MeV; intermediate
EBRT	CPT	77408	Radiation treatment delivery, 2 separate treatment areas, 3 or more ports on a single treatment area, use of multiple blocks; 6-10 MeV
EBRT	CPT	77409	Radiation treatment delivery, 2 separate treatment areas, 3 or more ports on a single treatment area, use of multiple blocks; 11-19 MeV
EBRT	CPT	77411	Radiation treatment delivery, 2 separate treatment areas, 3 or more ports on a single treatment area, use of multiple blocks; 20 MeV or greater
EBRT	CPT	77412	Radiation treatment delivery, =>1 MeV; complex
EBRT	CPT	77413	Radiation treatment delivery, 3 or more separate treatment areas, custom blocking, tangential ports, wedges, rotational beam, compensators, electron beam; 6-10 MeV
EBRT	CPT	77414	Radiation treatment delivery, 3 or more separate treatment areas, custom blocking, tangential ports, wedges, rotational beam, compensators, electron beam; 11-19 MeV
EBRT	CPT	77416	Radiation treatment delivery, 3 or more separate treatment areas, custom blocking, tangential ports, wedges, rotational beam, compensators, electron beam; 20 MeV or greater
Proton	CPT	77522	Proton treatment delivery; simple, with compensation
Proton	CPT	77523	Proton treatment delivery; intermediate
Proton	CPT	77525	Proton treatment delivery; complex
3DCRT	CPT	4165F	3-dimensional conformal radiotherapy (3DCRT-CRT) or intensity modulated radiation therapy (IMRT) received (PRCA)
3DCRT	CPT	4181F	Conformal radiation therapy received (NMA-No Measure Associated)
Proton	CPT	77520	Proton treatment delivery; simple, without compensation
EBRT	HCPCS	G6003	Radiation treatment delivery, single treatment area, single port or parallel opposed ports, simple blocks or no blocks: up to 5 mev
EBRT	HCPCS	G6004	Radiation treatment delivery, single treatment area, single port or parallel opposed ports, simple blocks or no blocks: 6-10 mev
EBRT	HCPCS	G6005	Radiation treatment delivery, single treatment area, single port or parallel opposed ports, simple blocks or no blocks: 11-19 mev
EBRT	HCPCS	G6006	Radiation treatment delivery, single treatment area, single port or parallel opposed ports, simple blocks or no blocks: 20 mev or greater
EBRT	HCPCS	G6007	Radiation treatment delivery, 2 separate treatment areas, 3 or more ports on a single treatment area, use of multiple blocks: up to 5 mev
EBRT	HCPCS	G6008	Radiation treatment delivery, 2 separate treatment areas, 3 or more ports on a single treatment area, use of multiple blocks: 6-10 mev
EBRT	HCPCS	G6009	Radiation treatment delivery, 2 separate treatment areas, 3 or more ports on a single treatment area, use of multiple blocks: 11-19 mev

© 2023 Roberts TJ et al. *JAMA Network Open.*

EBRT	HCPCS	G6010	Radiation treatment delivery, 2 separate treatment areas, 3 or more ports on a single treatment area, use of multiple blocks: 20 mev or greater
EBRT	HCPCS	G6011	Radiation treatment delivery, 3 or more separate treatment areas, custom blocking, tangential ports, wedges, rotational beam, compensators, electron beam; up to 5 mev
EBRT	HCPCS	G6012	Radiation treatment delivery, 3 or more separate treatment areas, custom blocking, tangential ports, wedges, rotational beam, compensators, electron beam; 6-10 mev
EBRT	HCPCS	G6013	Radiation treatment delivery, 3 or more separate treatment areas, custom blocking, tangential ports, wedges, rotational beam, compensators, electron beam; 11-19 mev
EBRT	HCPCS	G6014	Radiation treatment delivery, 3 or more separate treatment areas, custom blocking, tangential ports, wedges, rotational beam, compensators, electron beam; 20 mev or greater
EBRT	HCPCS	G6017	Intra-fraction localization and tracking of target or patient motion during delivery of radiation therapy (e.g., 3DCRT positional tracking, gating, 3DCRT surface tracking), each fraction of treatment
EBRT	ICD9	92.24	Teleradiotherapy Using Photons
EBRT	ICD9	92.25	Teleradiotherapy Using Electrons
EBRT	ICD9	92.26	Teleradiotherapy Of Other Particulate Radiation
EBRT	ICD9	92.29	Other Radiotherapeutic Procedure
EBRT	ICD10	D7050ZZ	Beam Radiation of Thorax Lymphatics using Photons <1 MeV
EBRT	ICD10	D7051ZZ	Beam Radiation of Thorax Lymphatics using Photons 1 - 10 MeV
EBRT	ICD10	D7052ZZ	Beam Radiation of Thorax Lymphatics using Photons >10 MeV
EBRT	ICD10	D7053Z0	Beam Radiation of Thorax Lymphatics using Electrons, Intraop
EBRT	ICD10	D7053ZZ	Beam Radiation of Thorax Lymphatics using Electrons
Proton	ICD10	D7054ZZ	Beam Radiation of Thorax Lymphatics using Heavy Particles
EBRT	ICD10	D7055ZZ	Beam Radiation of Thorax Lymphatics using Neutrons
EBRT	ICD10	DB000ZZ	Beam Radiation of Trachea using Photons <1 MeV
EBRT	ICD10	DB001ZZ	Beam Radiation of Trachea using Photons 1 - 10 MeV
EBRT	ICD10	DB002ZZ	Beam Radiation of Trachea using Photons >10 MeV
EBRT	ICD10	DB003Z0	Beam Radiation of Trachea using Electrons, Intraoperative
EBRT	ICD10	DB003ZZ	Beam Radiation of Trachea using Electrons
Proton	ICD10	DB004ZZ	Beam Radiation of Trachea using Heavy Particles
EBRT	ICD10	DB005ZZ	Beam Radiation of Trachea using Neutrons
EBRT	ICD10	DB010ZZ	Beam Radiation of Bronchus using Photons <1 MeV
EBRT	ICD10	DB011ZZ	Beam Radiation of Bronchus using Photons 1 - 10 MeV
EBRT	ICD10	DB012ZZ	Beam Radiation of Bronchus using Photons >10 MeV
EBRT	ICD10	DB013Z0	Beam Radiation of Bronchus using Electrons, Intraoperative
EBRT	ICD10	DB013ZZ	Beam Radiation of Bronchus using Electrons
Proton	ICD10	DB014ZZ	Beam Radiation of Bronchus using Heavy Particles
EBRT	ICD10	DB015ZZ	Beam Radiation of Bronchus using Neutrons
EBRT	ICD10	DB020ZZ	Beam Radiation of Lung using Photons <1 MeV
EBRT	ICD10	DB021ZZ	Beam Radiation of Lung using Photons 1 - 10 MeV
EBRT	ICD10	DB022ZZ	Beam Radiation of Lung using Photons >10 MeV
EBRT	ICD10	DB023Z0	Beam Radiation of Lung using Electrons, Intraoperative
EBRT	ICD10	DB023ZZ	Beam Radiation of Lung using Electrons
Proton	ICD10	DB024ZZ	Beam Radiation of Lung using Heavy Particles (Protons,Ions)
EBRT	ICD10	DB025ZZ	Beam Radiation of Lung using Neutrons
EBRT	ICD10	DB050ZZ	Beam Radiation of Pleura using Photons <1 MeV
EBRT	ICD10	DB051ZZ	Beam Radiation of Pleura using Photons 1 - 10 MeV
EBRT	ICD10	DB052ZZ	Beam Radiation of Pleura using Photons >10 MeV

© 2023 Roberts TJ et al. *JAMA Network Open.*

EBRT	ICD10	DB053Z0	Beam Radiation of Pleura using Electrons, Intraoperative
EBRT	ICD10	DB053ZZ	Beam Radiation of Pleura using Electrons
Proton	ICD10	DB054ZZ	Beam Radiation of Pleura using Heavy Particles
EBRT	ICD10	DB055ZZ	Beam Radiation of Pleura using Neutrons
EBRT	ICD10	DW020ZZ	Beam Radiation of Chest using Photons <1 MeV
EBRT	ICD10	DW021ZZ	Beam Radiation of Chest using Photons 1 - 10 MeV
EBRT	ICD10	DW022ZZ	Beam Radiation of Chest using Photons >10 MeV
EBRT	ICD10	DW023Z0	Beam Radiation of Chest using Electrons, Intraoperative
EBRT	ICD10	DW023ZZ	Beam Radiation of Chest using Electrons
Proton	ICD10	DW024ZZ	Beam Radiation of Chest using Heavy Particles (Protons,Ions)
EBRT	ICD10	DW025ZZ	Beam Radiation of Chest using Neutrons

© 2023 Roberts TJ et al. *JAMA Network Open.*

eTable 5. Billing Codes for Colorectal Cancer Resection Surgeries

CPT Code	Definition
44140	Colectomy, partial; with anastomosis
44141	Colectomy, partial; with skin level cecostomy or colostomy
44143	Colectomy, partial; with end colostomy and closure of distal segment (Hartman type procedure)
44144	Colectomy, partial; with resection, with colostomy or ileostomy and creation of mucofistula
44145	Colectomy, partial; with coloproctostomy (low pelvic anastomosis)
44146	Colectomy, partial; with coloproctostomy (low pelvic anastomosis), with colostomy
44147	Colectomy, partial; abdominal and transanal approach
44150	Colectomy, total, abdominal, without proctectomy; with ileostomy or ileoproctostomy
44151	Colectomy, total, abdominal, without proctectomy; with continent ileostomy
44155	Colectomy, total, abdominal, with proctectomy; with ileostomy
44156	Colectomy, total, abdominal, with proctectomy; with continent ileostomy
44157	Colectomy, total, abdominal, without proctectomy; with ileoanal anastomosis, includes loop ileostomy, and rectal mucosectomy, when performed
44158	Colectomy, total, abdominal, without proctectomy; with ileoanal anastomosis, creation of ileal reservoir (S or J), includes loop ileostomy, and rectal mucosectomy, when performed
44160	Colectomy, partial; with removal of terminal ileum with ileocolostomy
44204	Laparoscopic, surgical; colectomy, partial, with anastomosis
44205	Laparoscopic, surgical; colectomy, partial, with removal of terminal ileum with ileocolostomy
44206	Laparoscopic, surgical; colectomy, partial, with end colostomy and closure of distal segment (Hartman type procedure)
44207	Laparoscopic, surgical; colectomy, partial, with anastomosis, with coloproctostomy (low pelvic anastomosis)
44208	Laparoscopic, surgical; colectomy, partial, with anastomosis, with coloproctostomy (low pelvic anastomosis) with colostomy
44210	Laparoscopic, surgical; colectomy, total, abdominal, without proctectomy, with ileostomy or ileoproctostomy
44211	Laparoscopic, surgical; colectomy, total, abdominal, with proctectomy, with ileoanal anastomosis, creation of ileal reservoir (S or J), with loop ileostomy, includes rectal mucosectomy, when performed
44212	Laparoscopic, surgical; colectomy, total, abdominal, with proctectomy, with ileostomy
44213	Laparoscopic, surgical; mobilization (take-down) of splenic flexure performed in conjunction with partial colectomy
45111	Proctectomy; partial resection of rectum, transabdominal approach
45112	Proctectomy, combined abdominoperineal, pull-through procedure (eg, colo-anal anastomosis)
45113	Proctectomy, partial, with rectal mucosectomy, ileoanal anastomosis, creation of ileal reservoir (S or J), with or without loop ileostomy
45114	Proctectomy, partial, with anastomosis; abdominal and transsacral approach
45119	Proctectomy, combined abdominoperineal pull-through procedure (eg, colo-anal anastomosis), with creation of colonic reservoir (eg, J-pouch), with diverting enterostomy when performed

© 2023 Roberts TJ et al. *JAMA Network Open.*

eTable 6. Billing Codes for Molecular Tests

CPT Code	Description	Immunohistochemistry (IHC)	Multigene Panels
81201	APC (adenomatous polyposis coli) (eg, familial adenomatosis polyposis [FAP], attenuated FAP) gene analysis; full gene sequence		
81202	APC (adenomatous polyposis coli) (eg, familial adenomatosis polyposis [FAP], attenuated FAP) gene analysis; known familial variants		
81203	APC (adenomatous polyposis coli) (eg, familial adenomatosis polyposis [FAP], attenuated FAP) gene analysis; duplication/deletion variants		
81210	BRAF (B-Raf proto-oncogene, serine/threonine kinase) (eg, colon cancer, melanoma), gene analysis, V600 variant(s)		
81235	EGFR (epidermal growth factor receptor) (eg, non-small cell lung cancer) gene analysis, common variants (eg, exon 19 LREA deletion, L858R, T790M, G719A, G719S, L861Q)		
81275	KRAS (Kirsten rat sarcoma viral oncogene homolog) (eg, carcinoma) gene analysis; variants in exon 2 (eg, codons 12 and 13)		
81276	KRAS (Kirsten rat sarcoma viral oncogene homolog) (eg, carcinoma) gene analysis; additional variant(s) (eg, codon 61, codon 146)		
81288	MLH1 (mutL homolog 1, colon cancer, nonpolyposis type 2) (eg, hereditary non-polyposis colorectal cancer, Lynch syndrome) gene analysis; promoter methylation analysis		
81292	MLH1 (mutL homolog 1, colon cancer, nonpolyposis type 2) (eg, hereditary non-polyposis colorectal cancer, Lynch syndrome) gene analysis; full sequence analysis		
81293	MLH1 (mutL homolog 1, colon cancer, nonpolyposis type 2) (eg, hereditary non-polyposis colorectal cancer, Lynch syndrome) gene analysis; known familial variants		
81294	MLH1 (mutL homolog 1, colon cancer, nonpolyposis type 2) (eg, hereditary non-polyposis colorectal cancer, Lynch syndrome) gene analysis; duplication/deletion variants		
81295	MSH2 (mutS homolog 2, colon cancer, nonpolyposis type 1) (eg, hereditary non-polyposis colorectal cancer, Lynch syndrome) gene analysis; full sequence analysis		
81296	MSH2 (mutS homolog 2, colon cancer, nonpolyposis type 1) (eg, hereditary non-polyposis colorectal cancer, Lynch syndrome) gene analysis; known familial variants		
81297	MSH2 (mutS homolog 2, colon cancer, nonpolyposis type 1) (eg, hereditary non-polyposis colorectal cancer, Lynch syndrome) gene analysis; duplication/deletion variants		
81298	MSH6 (mutS homolog 6 [E. coli]) (eg, hereditary non-polyposis colorectal cancer, Lynch syndrome) gene analysis; full sequence analysis		
81299	MSH6 (mutS homolog 6 [E. coli]) (eg, hereditary non-polyposis colorectal cancer, Lynch syndrome) gene analysis; known familial variants		
81300	MSH6 (mutS homolog 6 [E. coli]) (eg, hereditary non-polyposis colorectal cancer, Lynch syndrome) gene analysis; duplication/deletion variants		

© 2023 Roberts TJ et al. *JAMA Network Open.*

81301	Microsatellite instability analysis (eg, hereditary non-polyposis colorectal cancer, Lynch syndrome) of markers for mismatch repair deficiency (eg, BAT25, BAT26), includes comparison of neoplastic and normal tissue, if performed		
81311	NRAS (neuroblastoma RAS viral [v-ras] oncogene homolog) (eg, colorectal carcinoma), gene analysis, variants in exon 2 (eg, codons 12 and 13) and exon 3 (eg, codon 61)		
81317	PMS2 (postmeiotic segregation increased 2 [S. cerevisiae]) (eg, hereditary non-polyposis colorectal cancer, Lynch syndrome) gene analysis; full sequence analysis		
81318	PMS2 (postmeiotic segregation increased 2 [S. cerevisiae]) (eg, hereditary non-polyposis colorectal cancer, Lynch syndrome) gene analysis; known familial variants		
81319	PMS2 (postmeiotic segregation increased 2 [S. cerevisiae]) (eg, hereditary non-polyposis colorectal cancer, Lynch syndrome) gene analysis; duplication/deletion variants		
81400	MOLECULAR PATHOLOGY PROCEDURE LEVEL 1		
81401	MOLECULAR PATHOLOGY PROCEDURE LEVEL 2		
81402	MOLECULAR PATHOLOGY PROCEDURE LEVEL 3		
81403	MOLECULAR PATHOLOGY PROCEDURE LEVEL 4		
81404	MOLECULAR PATHOLOGY PROCEDURE LEVEL 5		
81405	MOLECULAR PATHOLOGY PROCEDURE LEVEL 6		
81406	MOLECULAR PATHOLOGY PROCEDURE LEVEL 7		
81407	Molecular pathology procedure, Level 8		
81408	MOLECULAR PATHOLOGY PROCEDURE LEVEL 9		
81415	Exome (eg, unexplained constitutional or heritable disorder or syndrome); sequence analysis		1
81416	Exome (eg, unexplained constitutional or heritable disorder or syndrome); sequence analysis, each comparator exome (eg, parents, siblings) (List separately in addition to code for primary procedure)		1
81417	Exome (eg, unexplained constitutional or heritable disorder or syndrome); re-evaluation of previously obtained exome sequence (eg, updated knowledge or unrelated condition/syndrome)		1
81425	Genome (eg, unexplained constitutional or heritable disorder or syndrome); sequence analysis		1
81426	Genome (eg, unexplained constitutional or heritable disorder or syndrome); sequence analysis, each comparator genome (eg, parents, siblings) (List separately in addition to code for primary procedure)		1
81427	Genome (eg, unexplained constitutional or heritable disorder or syndrome); re-evaluation of previously obtained genome sequence (eg, updated knowledge or unrelated condition/syndrome)		1
81432	Hereditary breast cancer-related disorders (eg, hereditary breast cancer, hereditary ovarian cancer, hereditary endometrial cancer); genomic sequence analysis panel, must include sequencing of at least 10 genes, always including BRCA1, BRCA2, CDH1, MLH1, MSH2, MSH6, PALB2, PTEN, STK11, and TP53		1

© 2023 Roberts TJ et al. *JAMA Network Open.*

81433	Hereditary breast cancer-related disorders (eg, hereditary breast cancer, hereditary ovarian cancer, hereditary endometrial cancer); duplication/deletion analysis panel, must include analyses for BRCA1, BRCA2, MLH1, MSH2, and STK11		1
81435	Hereditary colon cancer disorders (eg, Lynch syndrome, PTEN hamartoma syndrome, Cowden syndrome, familial adenomatosis polyposis); genomic sequence analysis panel, must include sequencing of at least 10 genes, including APC, BMPR1A, CDH1, MLH1, MSH2, MSH6, MUTYH, PTEN, SMAD4, and STK11		1
81436	Hereditary colon cancer disorders (eg, Lynch syndrome, PTEN hamartoma syndrome, Cowden syndrome, familial adenomatosis polyposis); duplication/deletion analysis panel, must include analysis of at least 5 genes, including MLH1, MSH2, EPCAM, SMAD4, and STK11		1
81445	Targeted genomic sequence analysis panel, solid organ neoplasm, DNA analysis, and RNA analysis when performed, 5-50 genes (eg, ALK, BRAF, CDKN2A, EGFR, ERBB2, KIT, KRAS, NRAS, MET, PDGFRA, PDGFRB, PGR, PIK3CA, PTEN, RET), interrogation for sequence variants and copy number variants or rearrangements, if performed		1
81455	Targeted genomic sequence analysis panel, solid organ or hematolymphoid neoplasm, DNA analysis, and RNA analysis when performed, 51 or greater genes (eg, ALK, BRAF, CDKN2A, CEBPA, DNMT3A, EGFR, ERBB2, EZH2, FLT3, IDH1, IDH2, JAK2, KIT, KRAS, MLL, NPM1, NRAS, MET, NOTCH1, PDGFRA, PDGFRB, PGR, PIK3CA, PTEN, RET), interrogation for sequence variants and copy number variants or rearrangements, if performed		1
81479	Unlisted molecular pathology procedure		
81504	Oncology (tissue of origin), microarray gene expression profiling of > 2000 genes, utilizing formalin-fixed paraffin-embedded tissue, algorithm reported as tissue similarity scores		1
81525	Oncology (colon), mRNA, gene expression profiling by real-time RT-PCR of 12 genes (7 content and 5 housekeeping), utilizing formalin-fixed paraffin-embedded tissue, algorithm reported as a recurrence score		1
81540	Oncology (tumor of unknown origin), mRNA, gene expression profiling by real-time RT-PCR of 92 genes (87 content and 5 housekeeping) to classify tumor into main cancer type and subtype, utilizing formalin-fixed paraffin-embedded tissue, algorithm reported as a probability of a predicted main cancer type and subtype		1
81599	Unlisted multianalyte assay with algorithmic analysis		
83890	Molecular diagnostics; molecular isolation or extraction, each nucleic acid type (ie, DNA or RNA)		
83891	Molecular diagnostics; isolation or extraction of highly purified nucleic acid, each nucleic acid type (ie, DNA or RNA)		
83892	Molecular diagnostics; enzymatic digestion, each enzyme treatment		

© 2023 Roberts TJ et al. *JAMA Network Open.*

83893	Molecular diagnostics; dot/slot blot production, each nucleic acid preparation		
83894	Molecular diagnostics; separation by gel electrophoresis (eg, agarose, polyacrylamide), each nucleic acid preparation		
83896	Molecular diagnostics; nucleic acid probe, each		
83897	Molecular diagnostics; nucleic acid transfer (eg, Southern, Northern), each nucleic acid preparation		
83898	Molecular diagnostics; amplification, target, each nucleic acid sequence		
83900	Molecular diagnostics; amplification, target, multiplex, first 2 nucleic acid sequences		
83901	Molecular diagnostics; amplification, target, multiplex, each additional nucleic acid sequence beyond 2 (List separately in addition to code for primary procedure)		
83902	Molecular diagnostics; reverse transcription		
83903	Molecular diagnostics; mutation scanning, by physical properties (eg, single strand conformational polymorphisms [SSCP], heteroduplex, denaturing gradient gel electrophoresis [DGGE], RNA'ase A), single segment, each		
83904	Molecular diagnostics; mutation identification by sequencing, single segment, each segment		
83905	Molecular diagnostics; mutation identification by allele specific transcription, single segment, each segment		
83906	Molecular diagnostics; mutation identification by allele specific translation, single segment, each segment		
83907	Molecular diagnostics; lysis of cells prior to nucleic acid extraction (eg, stool specimens, paraffin embedded tissue), each specimen		
83908	Molecular diagnostics; amplification, signal, each nucleic acid sequence		
83909	Molecular diagnostics; separation and identification by high resolution technique (eg, capillary electrophoresis), each nucleic acid preparation		
83912	Molecular diagnostics; interpretation and report		
83913	Molecular diagnostics; RNA stabilization		
83914	Mutation identification by enzymatic ligation or primer extension, single segment, each segment (eg, oligonucleotide ligation assay [OLA], single base chain extension [SBCE], or allele-specific primer extension [ASPE])		
83950	Oncoprotein; HER-2/neu		
88271	Molecular cytogenetics; DNA probe, each (eg, FISH)		
88272	Molecular cytogenetics; chromosomal in situ hybridization, analyze 3-5 cells (eg, for derivatives and markers)		
88273	Molecular cytogenetics; chromosomal in situ hybridization, analyze 10-30 cells (eg, for microdeletions)		
88274	Molecular cytogenetics; interphase in situ hybridization, analyze 25-99 cells		
88275	Molecular cytogenetics; interphase in situ hybridization, analyze 100-300 cells		
88291	Cytogenetics and molecular cytogenetics, interpretation and report		
88299	Unlisted cytogenetic study		

© 2023 Roberts TJ et al. *JAMA Network Open.*

88341	Immunohistochemistry or immunocytochemistry, per specimen; each additional single antibody stain procedure (List separately in addition to code for primary procedure)	1	
88342	Immunohistochemistry or immunocytochemistry, per specimen; initial single antibody stain procedure	1	
88343	Immunohistochemistry or immunocytochemistry, each separately identifiable antibody per block, cytologic preparation, or hematologic smear; each additional separately identifiable antibody per slide (List separately in addition to code for primary procedure)	1	
88344	Immunohistochemistry or immunocytochemistry, per specimen; each multiplex antibody stain procedure	1	
88360	Morphometric analysis, tumor immunohistochemistry (eg, Her-2/neu, estrogen receptor/progesterone receptor), quantitative or semiquantitative, per specimen, each single antibody stain procedure; manual	1	
88361	Morphometric analysis, tumor immunohistochemistry (eg, Her-2/neu, estrogen receptor/progesterone receptor), quantitative or semiquantitative, per specimen, each single antibody stain procedure; using computer-assisted technology	1	
88363	Examination and selection of retrieved archival (ie, previously diagnosed) tissue(s) for molecular analysis (eg, KRAS mutational analysis)		
88364	In situ hybridization (eg, FISH), per specimen; each additional single probe stain procedure (List separately in addition to code for primary procedure)		
88365	In situ hybridization (eg, FISH), per specimen; initial single probe stain procedure		
88366	In situ hybridization (eg, FISH), per specimen; each multiplex probe stain procedure		
88367	Morphometric analysis, in situ hybridization (quantitative or semi-quantitative), using computer-assisted technology, per specimen; initial single probe stain procedure		
88368	Morphometric analysis, in situ hybridization (quantitative or semi-quantitative), manual, per specimen; initial single probe stain procedure		
88369	Morphometric analysis, in situ hybridization (quantitative or semi-quantitative), manual, per specimen; each additional single probe stain procedure (List separately in addition to code for primary procedure)		
88373	Morphometric analysis, in situ hybridization (quantitative or semi-quantitative), using computer-assisted technology, per specimen; each additional single probe stain procedure (List separately in addition to code for primary procedure)		
88374	Morphometric analysis, in situ hybridization (quantitative or semi-quantitative), using computer-assisted technology, per specimen; each multiplex probe stain procedure		
88377	Morphometric analysis, in situ hybridization (quantitative or semi-quantitative), manual, per specimen; each multiplex probe stain procedure		
88384	Array-based evaluation of multiple molecular probes; 11 through 50 probes		1
88385	Array-based evaluation of multiple molecular probes; 51 through 250 probes		1

© 2023 Roberts TJ et al. *JAMA Network Open.*

88386	Array-based evaluation of multiple molecular probes; 251 through 500 probes		1
0007M	Oncology (gastrointestinal neuroendocrine tumors), real-time PCR expression analysis of 51 genes, utilizing whole peripheral blood, algorithm reported as a nomogram of tumor disease index		1
0013U	Oncology (solid organ neoplasia), gene rearrangement detection by whole genome next-generation sequencing, DNA, fresh or frozen tissue or cells, report of specific gene rearrangement(s)		1
0019U	Oncology, RNA, gene expression by whole transcriptome sequencing, formalin-fixed paraffin embedded tissue or fresh frozen tissue, predictive algorithm reported as potential targets for therapeutic agents		1
0022U	Targeted genomic sequence analysis panel, non-small cell lung neoplasia, DNA and RNA analysis, 23 genes, interrogation for sequence variants and rearrangements, reported as presence/absence of variants and associated therapy(ies) to consider		1
0036U	Exome (ie, somatic mutations), paired formalin-fixed paraffin-embedded tumor tissue and normal specimen, sequence analyses		1
0037U	Targeted genomic sequence analysis, solid organ neoplasm, DNA analysis of 324 genes, interrogation for sequence variants, gene copy number amplifications, gene rearrangements, microsatellite instability and tumor mutational burden		1
0048U	Oncology (solid organ neoplasia), DNA, targeted sequencing of protein-coding exons of 468 cancer-associated genes, including interrogation for somatic mutations and microsatellite instability, matched with normal specimens, utilizing formalin-fixed paraffin-embedded tumor tissue, report of clinically significant mutation(s)		1
0057U	Oncology (solid organ neoplasia), mRNA, gene expression profiling by massively parallel sequencing for analysis of 51 genes, utilizing formalin-fixed paraffin-embedded tissue, algorithm reported as a normalized percentile rank		1
0069U	Oncology (colorectal), microRNA, RT-PCR expression profiling of miR-31-3p, formalin-fixed paraffin-embedded tissue, algorithm reported as an expression score		
0094U	Genome (eg, unexplained constitutional or heritable disorder or syndrome), rapid sequence analysis		1
0101U	Hereditary colon cancer disorders		1
0104U	Hereditary pan cancer (eg, hereditary breast and ovarian cancer, hereditary endometrial cancer, hereditary colorectal cancer), genomic sequence analysis panel utilizing a combination of NGS, Sanger, MLPA, and array CGH, with MRNA analytics to resolve variants of unknown significance when indicated (32 genes [sequencing and deletion/duplication], EPCAM and GREM1 [deletion/duplication only])		1
0111U	Oncology (colon cancer), targeted KRAS (codons 12, 13, and 61) and NRAS (codons 12, 13, and 61) gene analysis utilizing formalin-fixed paraffin-embedded tissue		

© 2023 Roberts TJ et al. *JAMA Network Open.*

0130U	Hereditary colon cancer disorders (eg, Lynch syndrome, PTEN hamartoma syndrome, Cowden syndrome, familial adenomatosis polyposis), targeted mRNA sequence analysis panel (APC, CDH1, CHEK2, MLH1, MSH2, MSH6, MUTYH, PMS2, PTEN, and TP53) (List separately in addition to code for primary procedure)		1
0134U	Hereditary pan cancer (eg, hereditary breast and ovarian cancer, hereditary endometrial cancer, hereditary colorectal cancer), targeted mRNA sequence analysis panel (18 genes) (List separately in addition to code for primary procedure)		1

Multigene panels were defined as panels that included testing for at least 5 genes.

© 2023 Roberts TJ et al. *JAMA Network Open.*

eTable 7. *ICD-10* Codes for Colorectal Cancer Categorized by Site

ICD-10 Code	Description	Side
C180	Malignant neoplasm of cecum	right sided
C181	Malignant neoplasm of appendix	right sided
C182	Malignant neoplasm of ascending colon	right sided
C183	Malignant neoplasm of hepatic flexure	right sided
C184	Malignant neoplasm of transverse colon	right sided
C185	Malignant neoplasm of splenic flexure	left sided
C186	Malignant neoplasm of descending colon	left sided
C187	Malignant neoplasm of sigmoid colon	left sided
C188	Malignant neoplasm of overlapping sites of colon	NOS
C189	Malignant neoplasm of colon, unspecified	NOS
C19	Malignant neoplasm of rectosigmoid junction	rectal
C20	Malignant neoplasm of rectum	rectal

© 2023 Roberts TJ et al. *JAMA Network Open.*

eTable 8. Full Regression Results

		Any Molecular Testing				Targeted Therapy Use			
		NSCLC		Colorectal Cancer		NSCLC		Colorectal Cancer	
		Unadjusted rate	Adjusted PP diff. (95% CI)	Unadjusted rate	Adjusted PP diff. (95% CI)	Unadjusted rate	Adjusted PP diff. (95% CI)	Unadjusted rate	Adjusted PP diff. (95% CI)
**Year and quarter**			0.5 (0.4 to 0.5)		1.0 (0.9 to 1.1)		-0.3 (-0.4 to -0.3)		-0.01 (-0.07 to 0.05)
**Age**	65-69 (ref.)	71.3		47.7		9.0		7.1	
	70-74	81.7	9.0 (8.4 to 9.6)	58.1	8.8 (7.5 to 10.1)	7.1	-1.0 (-1.6 to -0.4)	4.3	-2.5 (-3.2 to -1.8)
	75-59	83.4	10.3 (9.6 to 11.0)	58.6	8.8 (7.4 to 10.2)	7.6	-0.2 (-0.8 to 0.4)	3.3	-3.4 (-4.2 to -2.7)
	80-84	83.7	10.6 (9.8 to 11.4)	57.6	7.5 (5.9 to 9.1)	11.9	3.4 (2.6 to 4.1)	2.8	-3.8 (-4.7 to -2.9)
	≥ 85	83.3	10.0 (9.0 to 11.1)	52.6	2.5 (0.7 to 4.2)	20.3	10.7 (9.6 to 11.7)	2.0	-4.4 (-5.5 to -3.4)
**Sex**	Female (ref.)	79.7		54.8		12.2		4.0	
	Male	79.0	-1.4 (-1.9 to -1.0)	53.1	-2.0 (-3.0 to -1.1)	5.9	-5.8 (-6.3 to -5.4)	5.4	1.2 (0.7 to 1.8)
**Race**	White, non-Hispanic (ref.)	80.2		54.7		7.4		4.6	
	Black	74.6	-2.7 (-3.7 to -1.8)	51.0	-1.9 (-3.8 to -0.1)	7.5	1.0 (0.1 to 1.9)	5.2	0.2 (-0.9 to 1.2)
	Hispanic	73.6	-2.0 (-4.6 to 0.5)	44.6	-4.7 (-8.7 to -0.7)	20.8	11.9 (9.8 to 14.1)	4.8	0.5 (-2.6 to 1.6)
	Asian	75.5	-1.2 (-3.0 to 0.5)	45.3	-5.6 (-8.9 to -2.2)	46.6	34.7 (33.2 to 36.1)	4.5	-0.4 (-2.2 to 1.3)
	Other	73.5	-3.5 (-4.8 to -2.1)	51.4	-0.9 (-3.3 to 1.5)	22.6	14.0 (12.7 to 15.3)	6.5	0.7 (-0.7 to 2.1)
**Urban/ rural**	Urban (ref.)	79.3		53.8		10.0		4.9	
	Large rural	79.2	0.4 (-0.4 to 1.3)	52.2	-1.2 (-2.9 to 0.4)	6.1	-0.4 (-1.2 to 0.4)	3.8	-1.1 (-2.0 to -0.1)
	Small rural	80.2	1.5 (0.4 to 2.6)	55.3	1.4 (-0.7 to 3.4)	5.3	-1.1 (-2.1 to -0.1)	4.7	-0.1 (-1.2 to 1.1)
	Isolated	80.3	1.9 (0.7 to 3.1)	56.3	2.2 (-0.2 to 4.6)	6.7	-0.5 (-1.7 to 0.6)	4.3	-0.6 (-1.9 to 0.8)
**Median income***	≥ $80,000 (ref.)	80.8		55.4		13.5		4.8	
	$60,000 - $80,000	79.6	-0.6 (-1.5 to 0.2)	55.4	1.0 (-0.8 to 2.7)	10.4	-0.4 (-1.2 to 0.4)	4.6	-0.3 (-1.2 to 0.7)
	$40,000 – $60,000	79.5	-0.3 (-1.3 to 0.6)	53.7	0.9 (-0.9 to 2.8)	7.8	-1.5 (-2.3 to -0.6)	4.8	-0.1 (-1.1 to 0.9)
	< $40,000	77.6	-1.1 (-2.2 to 0.1)	51.7	0.7 (-1.7 to 3.0)	6.9	-2.1 (-3.2 to -1.1)	4.6	-0.3 (-1.6 to 1.0)
	Unknown	80.7	-0.6 (-5.4 to 4.2)	52.5	-6.6 (-16.3 to 3.1)	9.8	-2.4 (-7.0 to 2.1)	5.7	-3.0 (-8.5 to 2.5)
**High school graduation rate***	≥ 93% (ref.)	80.8		55.9		11.5		4.6	
	89% - 93%	79.5	-1.1 (-1.8 to -0.3)	55.2	-0.2 (-1.7 to 1.3)	8.4	-1.3 (-2.0 to -0.7)	4.6	0.02 (-0.8 to 0.9)
	83% – 89%	79.3	-1.1 (-1.9 to -0.2)	53.2	-1.6 (-3.3 to 0.1)	7.3	-1.5 (-2.3 to -0.8)	4.7	0.2 (-0.7 to 1.1)
	0% – 83%	77.4	-1.8 (-2.8 to -0.8)	51.0	-2.3 (-4.2 to -0.4)	8.9	-1.1 (-2.0 to -0.2)	4.9	0.3 (-0.8 to 1.3)
	Unknown	80.8	1.1 (-4.1 to 6.3)	53.6	7.5 (-2.8 to 17.8)	10.4	2.1 (-2.8 to 7.0)	6.4	4.3 (-1.6 to 10.2)
**Dual eligible**	No (ref.)	80.3		54.9		8.3		4.7	
	Yes	74.6	-3.9 (-4.6 to -3.2)	49.1	-4.2 (-5.6 to -2.8)	12.6	1.1 (0.5 to 1.8)	4.7	-0.3 (-1.1 to 0.4)
**Charlson score**	0 (ref.)	72.5		48.8		13.4		5.3	
	1	83.6	10.7 (10.1 to 11.3)	61.0	11.4 (10.1 to 12.7)	6.6	-5.6 (-6.2 to -5.0)	4.0	-0.8 (-1.6 to -0.1)
	2	82.8	9.7 (9.0 to 10.4)	59.9	10.2 (8.6 to 11.8)	6.0	-5.7 (-6.4 to -5.0)	4.1	-0.6 (-1.5 to 0.3)
	3+	83.9	10.4 (9.8 to 11.1)	59.8	10.0 (8.5 to 11.5)	5.9	-5.5 (-6.2 to -4.8)	3.5	-1.1 (-2.0 to -0.3)
**Practice type**	NCI Center (ref.)	78.9		56.9		16.6		5.0	
	Academic center	78.3	-1.3 (-3.7 to 1.1)	57.2	0.8 (-3.2 to 4.9)	9.1	-4.8 (-6.4 to -3.2)	6.1	1.2 (-0.4 to 2.7)
	Other hospital	79.3	-0.4 (-2.0 to 1.2)	54.2	-3.8 (-6.4 to -1.2)	8.2	-5.4 (-6.4 to -4.3)	4.8	-0.05 (-1.0 to 0.9)
	Large independent	80.4	-0.01 (-1.7 to 1.7)	53.7	-3.3 (-6.1 to -0.4)	6.9	-6.4 (-7.5 to -5.2)	4.4	-0.3 (-1.4 to 0.9)
	Small independent	78.1	-1.4 (-3.3 to 0.5)	45.1	-12.2 (-15.3 to -9.1)	8.5	-6.2 (-7.6 to -4.8)	3.6	-1.2 (-2.6 to 0.1)
**Primary tumor site**	Right-sided (ref.)			57.2				3.6	
	Left-sided			54.5	-0.8 (-2.4 to 0.9)			9.8	5.7 (4.8 to 6.6)
	Rectal			52.9	-3.7 (-4.9 to -2.4)			3.2	-0.6 (-1.4 to 0.1)
	NOS			52.0	-4.4 (-5.9 to -2.8)			6.2	2.6 (1.7 to 3.5)

PP=percentage point

© 2023 Roberts TJ et al. *JAMA Network Open.*

eTable 9. NSCLC Molecular Testing Sensitivity Analysis (Excluding IHC)

	Adjusted PP diff. (95% CI)	
**Year and quarter**	0.8%	(0.7 to 0.8)
**Age**		
65-69 (ref)		
70-74	5.6%	(4.8 to 6.4)
75-59	6.4%	(5.6 to 7.2)
80-84	8.7%	(7.7 to 9.6)
≥ 85	10.9%	(9.7 to 12.2)
**Sex**		
Female (ref)		
Male	-3.9%	(-4.5 to -3.3)
**Race**		
White, non-Hispanic (ref)		
Black	-0.9%	(-2.1 to 0.3)
Hispanic	1.0%	(-2.1 to 4.2)
Asian	5.7%	(3.5 to 7.8)
Other/Unknown	0.2%	(-1.5 to 1.8)
**Urban/rural**		
Urban (ref)		
Large Rural	0.2%	(-0.9 to 1.3)
Small Rural	0.5%	(-0.8 to 1.9)
Isolated	0.4%	(-1.2 to 1.9)
**Median income***		
≥ $80,000 (ref)		
$60,000 - $80,000	-1.5%	(-2.6 to -0.4)
$40,000 – $60,000	-2.1%	(-3.3 to -0.9)
< $40,000	-2.5%	(-4.0 to -1.0)
Unknown	-1.3%	(-7.3 to 4.6)
**High school graduation rate***		
≥ 93% (ref)		
89% - 93%	-0.9%	(-1.7 to 0.1)
83% – 89%	-2.1%	(-3.1 to -1.0)
0% – 83%	-2.9%	(-4.2 to -1.7)
Unknown	1.6%	(-4.8 to 8.0)
**Dual eligible**		
No (ref)		
Yes	-4.1%	(-5.0 to -3.2)
**Charlson score**		
0 (ref)		
1	2.5%	(1.8 to 3.2)
2	0.6%	(-0.3 to 1.5)
3+	-0.5%	(-1.3 to 0.3)
**Practice type**		
NCI center (ref)		
Academic Center	-6.7%	(-10.6 to -2.8)
Other Hospital	-1.6%	(-4.1 to 1.0)
Large Independent Practice	-0.5%	(-3.5 to 2.5)
Small Independent Practice	-2.5%	(-5.5 to 0.5)

PP = percentage point

© 2023 Roberts TJ et al. *JAMA Network Open.*

eTable 10. NSCLC Molecular Testing Sensitivity Analysis (Bevacizumab/Pemetrexed)

	Adjusted PP diff. (95% CI)	
**Year and quarter**	0.3%	(0.2 to 0.3)
**Age**		
65-69 (ref)		
70-74	6.6%	(5.6 to 7.5)
75-59	7.1%	(6.1 to 8.2)
80-84	5.3%	(4.0 to 6.6)
≥ 85	-2.5%	(-4.3 to -0.6)
**Sex**		
Female (ref)		
Male	1.2%	(0.5 to 2.0)
**Race**		
White, non-Hispanic (ref)		
Black	-1.4%	(-2.9 to 0.2)
Hispanic	1.4%	(-3.0 to 5.7)
Asian	-5.5%	(-8.5 to -2.5)
Other/Unknown	-4.9%	(-7.0 to -2.8)
**Urban/rural**		
Urban (ref)		
Large Rural	0.4%	(-1.0 to 1.7)
Small Rural	2.3%	(0.5 to 4.0)
Isolated	1.1%	(-0.9 to 3.1)
**Median income***		
≥ $80,000 (ref)		
$60,000 - $80,000	-1.3%	(-2.6 to 0.0)
$40,000 – $60,000	-0.5%	(-1.9 to 0.9)
< $40,000	-1.5%	(-3.3 to 0.3)
Unknown	-3.9%	(-11.8 to 4.0)
**High school graduation rate***		
≥ 93% (ref)		
89% - 93%	0.2%	(-1.0 to 1.3)
83% – 89%	-0.6%	(-1.8 to 0.7)
0% – 83%	-0.2%	(-1.6 to 1.3)
Unknown	3.7%	(-11.8 to 4.0)
**Dual eligible**		
No (ref)		
Yes	-3.7%	(-5.0 to -2.5)
**Charlson score**		
0 (ref)		
1	8.6%	(7.7 to 9.5)
2	8.0%	(6.9 to 9.2)
3+	8.3%	(7.2 to 9.4)
**Practice type**		
NCI center (ref)		
Academic Center	-1.8%	(-4.9 to 1.2)
Other Hospital	-0.7%	(-2.7 to 1.2)
Large Independent Practice	-0.4%	(-2.8 to 1.9)
Small Independent Practice	-4.3%	(-6.9 to -1.6)

PP = percentage point

eTable 11. CRC Molecular Testing Sensitivity Analysis (Excluding IHC)

	Adjusted PP diff. (95% CI)

© 2023 Roberts TJ et al. *JAMA Network Open.*

**Year and quarter**	0.6%	(0.6 to 0.7)
**Age**		
65-69 (ref)		
70-74	3.7%	(2.6 to 4.8)
75-59	3.2%	(2.1 to 4.4)
80-84	1.2%	(-0.1 to 2.6)
≥ 85	-1.8%	(-3.2 to 0.3)
**Sex**		
Female (ref)		
Male	0.5%	(-0.3 to 1.4)
**Race**		
White, non-Hispanic (ref)		
Black	-1.0%	(-2.5 to 0.6)
Hispanic	-2.4%	(-5.8 to 1.0)
Asian	-2.7%	(-5.5 to 0.2)
Other/Unknown	0.7%	(-1.3 to 2.70)
**Urban/rural**		
Urban (ref)		
Large Rural	-0.8%	(-2.2 to 0.6)
Small Rural	2.0%	(0.2 to 3.7)
Isolated	0.5%	(-1.6 to 2.5)
**Median income***		
≥ $80,000 (ref)		
$60,000 - $80,000	1.1%	(-0.4 to 2.6)
$40,000 – $60,000	1.4%	(-0.2 to 3.0)
< $40,000	1.3%	(0.7 to 3.3)
Unknown	-1.3%	(-9.5 to 6.9)
**High school graduation rate***		
≥ 93% (ref)		
89% - 93%	-0.4%	(-1.7 to 0.9)
83% – 89%	-0.7%	(-2.1 to 0.7)
0% – 83%	-1.0%	(-2.6 to 0.6)
Unknown	2.2%	(-6.6 to 10.9)
**Dual eligible**		
No (ref)		
Yes	-2.9%	(-4.1 to -1.8)
**Charlson score**		
0 (ref)		
1	5.2%	(4.1 to 6.2)
2	3.4%	(2.1 to 4.8)
3+	2.3%	(1.0 to 3.6)
**Practice type**		
NCI center (ref)		
Academic Center	-0.3%	(-3.7 to 3.0)
Other Hospital	0.0%	(-2.1 to 2.2)
Large Independent Practice	1.4%	(-1.2 to 4.1)
Small Independent Practice	-6.3%	(-9.1 to -3.5)
**Tumor location**		
Right-sided (ref)		
Left-sided	0.3%	(-1.1 to 1.7)
Rectal	-11.8%	(-12.9 to -10.7)
Not specified	-2.7%	(-4.0 to -1.3)

PP = percentage point

eTable 12. NSCLC Targeted Therapy Sensitivity Analysis (2017-2019)

	Adjusted PP diff. (95% CI)	
**Year and quarter**	-0.1%	(-0.21 to 0.0)
**Age**		

65-69 (ref)		
70-74	-0.7%	(-1.4 to 0.0)
75-59	-0.1%	(-0.9 to 0.6)
80-84	2.5%	(1.6 to 3.4)
≥ 85	7.5%	(6.3 to 8.8)
**Sex**		
Female (ref)		
Male	-5.3%	(-.8 to -4.7)
**Race**		
White, non-Hispanic (ref)		
Black	0.8%	(-0.3 to 1.9)
Hispanic	11.9%	(9.2 to 14.5)
Asian	35.9%	(34.1 to 37.7)
Other/Unknown	13.3%	(11.8 to 14.7)
**Urban/rural**		
Urban (ref)		
Large Rural	-0.5%	(-1.4 to 0.4)
Small Rural	-0.3%	(-1.6 to 0.9)
Isolated	-0.9%	(-2.2 to 0.5)
**Median income***		
≥ $80,000 (ref)		
$60,000 - $80,000	-0.5%	(-1.4 to 0.4)
$40,000 – $60,000	-2.1%	(-3.1 to -1.1)
< $40,000	-2.9%	(-4.2 to -1.6)
Unknown	-2.3%	(-7.7 to 3.1)
**High school graduation rate***		
≥ 93% (ref)		
89% - 93%	-1.2%	(-2.1 to -0.4)
83% – 89%	-1.5%	(2.5 to -0.4)
0% – 83%	-1.0%	(-2.1 to 0.0)
Unknown	2.0%	(-3.9 to 7.9)
**Dual eligible**		
No (ref)		
Yes	2.2%	(1.5 to 3.0)
**Charlson score**		
0 (ref)		
1	-5.4%	(-6.1 to -4.7)
2	-5.4%	(-6.3 to -4.6)
3+	-5.6%	(-6.4 to -4.8)
**Practice type**		
NCI center (ref)		
Academic Center	-5.7%	(-7.5 to -4.0)
Other Hospital	-6.2%	(-7.4 to -5.1)
Large Independent Practice	-7.0%	(-8.5 to -5.6)
Small Independent Practice	-6.4%	(-8.2 to -4.6)

PP = percentage point

© 2023 Roberts TJ et al. *JAMA Network Open.*

eTable 13. Variation Across Oncology Practices

	Molecular Testing		Targeted Therapy Use	
	**Colorectal Cancer**	**NSCLC**	**Colorectal Cancer**	**NSCLC**
**95%**	60.5	83.7	5.3	11.6
**75%**	55.5	80.4	4.7	9.4
**50%**	**53.0**	**79.7**	**4.7**	**9.0**
**25%**	52.1	78.0	4.6	8.5
**10%**	50.3	76.6	4.4	7.8
**5%**	48.8	75.2	4.3	7.3

© 2023 Roberts TJ et al. *JAMA Network Open.*

eFigure. Trends in Use of Erlotinib and Other Targeted Therapies

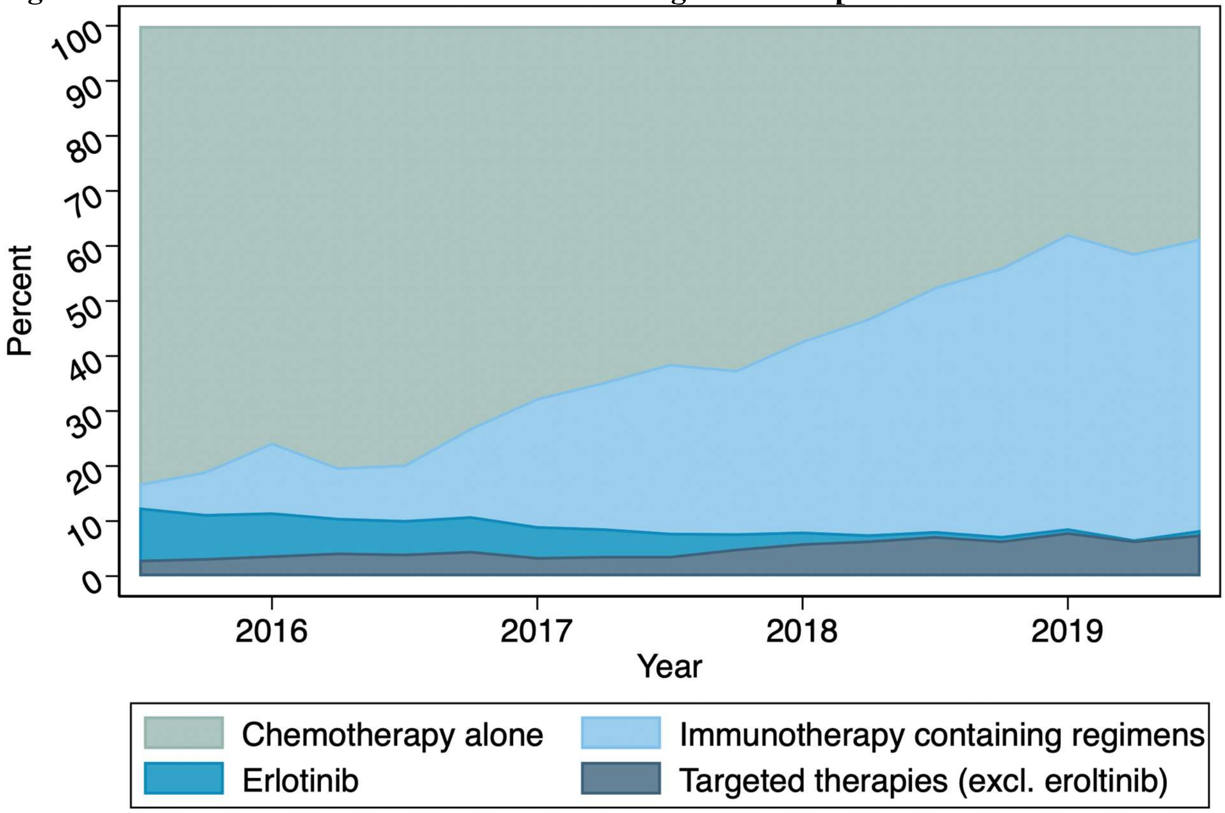

© 2023 Roberts TJ et al. *JAMA Network Open.*
