# Supplementary material for: Practice-Level Variation in Molecular Testing and Use of Targeted Therapy for Patients With Non–Small Cell Lung Cancer and Colorectal Cancer
Source: JAMA Netw Open. 2023 Apr 28;6(4):e2310809. doi: 10.1001/jamanetworkopen.2023.10809 (PMC10148196; doi:10.1001/jamanetworkopen.2023.10809)
Supplement: Supplement 2. — Data Sharing Statement [file jamanetwopen-e2310809-s002.pdf]

Data Sharing Statement

Roberts. Practice-Level Variation in Molecular Testing and Use of Targeted Therapy for Patients With Non-Small Cell Lung Cancer and Colorectal Cancer. *JAMA Netw Open*. Published April 28, 2023. doi:10.1001/jamanetworkopen.2023.10809

Data
Data available: No

Additional Information
Explanation for why data not available: Medicare claims
